# Effects of resistant starch consumption on anthropometric and serum parameters in adults with metabolic syndrome-related risks: a systematic review and meta-analysis

**DOI:** 10.3389/fnut.2025.1655664

**Published:** 2025-09-25

**Authors:** Ximing Lin, Zaizhen Li, Dongyuan Zheng, Ruofang Du, Ruikang Zhong, Changqing Lin, Hua Meng

**Affiliations:** ^1^Department of General Surgery and Obesity and Metabolic Center, China-Japan Friendship Hospital, Beijing, China; ^2^Medical College, Yanbian University, Yanbian, Jilin, China; ^3^Graduate School, Beijing University of Chinese Medicine, Beijing, China

**Keywords:** resistant starch, effects, metabolic syndrome-related risks, systematic review, meta-analysis

## Abstract

**Background:**

The effects of resistant starch (RS) consumption on anthropometric and serum biomarkers in adults with metabolic syndrome (MetS)-related risks, each component of which similarly increases the incidence of cardiovascular disease, have yielded inconclusive results when compared to anticipated outcomes. The heterogenous effects of RS type, delivery mode, participant characteristics, intervention conditions, and the quality of study design on the observed outcomes are considered to be insufficiently understood.

**Methods:**

A comprehensive search was conducted in five public databases and 30 previously published meta-analyses up to January 21, 2025, following the PRISMA guidelines. A total of 23 parallel or crossover randomized controlled trials were included for qualitative analysis via Cochrane Risk of Bias tool and the Jadad scale. Among, 19 studies were included for synthesizing effect sizes of changes in anthropometric parameters, glycemic and lipid profiles, inflammatory markers, and oxidative stress biomarkers using a random-effects model. Subgroup analysis was performed to explore contributes of heterogeneity. Sensitivity analysis and publication bias analysis were conducted.

**Results:**

RS consumption was associated with significant reductions in hip circumference (MD = −1.83 cm; 95% CI: −2.03 to −1.64), total cholesterol (MD = −0.20 mmol/L; 95% CI: −0.32 to −0.08), low-density lipoprotein cholesterol (MD = −0.11 mmol/L; 95% CI: −0.18 to −0.04), and improved superoxide dismutase levels (SMD = 0.29; 95% CI: 0.08–0.51). Waist circumference, fasting insulin, HOMA-IR, and TNF-α were reduced by RS with high heterogeneity yet. High quality of study design, participants with younger age and overweight, a supplement as delivery, a dose of up to 30 g/day, and lasting over 8 weeks partly influenced the effects.

**Conclusion:**

Steady effects of RS were observed on hip circumference, total cholesterol, low-density lipoprotein cholesterol, and superoxide dismutase in adults with MetS-related risks. For the intervention with RS, it is recommended that participants be younger and overweight, with a dosage of at least 30 g/day, and over a period of 8 weeks. Future studies should be designed with high methodological quality, with considerations of delivery mode, properties, as well as gut microbiome and metabolome.

**Systematic review registration:**

https://www.crd.york.ac.uk/PROSPERO/view/CRD420251014654 CRD420251014654.

## 1 Introduction

Metabolic syndrome (MetS) constitutes a major global public health challenge in adults, feeding into various chronic diseases, especially cardiovascular disease (CVD) (1). The common components of MetS consist of visceral obesity, hyperglycemia, hypertension, low high-density lipoprotein cholesterol (HDL-C), raised triglycerides (TG), according to Adult Treatment Panel III and International Diabetes Federation guidelines (2). Excessive body mass index (BMI) is considered as another component based on American Association of Clinical Endocrinologists guideline (3). Either each component of MetS or the combination together serves as high risk factors for the development of CVD (4). A 13-year prospective study has reported that either MetS or its single components increased similar incidence of cardiovascular events by 20–60% (5). Inflammation and oxidative stress are involved in the development from MetS to CVD (6). Therefore, the prompt treatment and management of MetS-related risks is essential for preventing cardiovascular complications and alleviating metabolic burden.

Resistant starch (RS) is characterized as a type of starch that remains undigested in the small intestine and undergoes fermentation in the large intestine by producing short-chain fatty acids (SCFAs) (7). Evidence has shown RS can improve cardiometabolic outcomes and attenuates MetS risk-related diseases, such as overweight or obesity (8), prediabetes or type 2 diabetes mellitus (T2DM) (9), hyperlipidemia (10) and non-alcoholic fatty liver disease (NAFLD) (11) to varying degrees. RS improves cardiometabolic function including lowered body weight (BW), BMI, body dimensions and fat composition (12), stabled blood pressure (13), improved glycemic control and insulin sensitivity (14, 15), lowered lipid profiles (10, 16), and reduced inflammation and oxidative stress levels (17, 18). Therefore, RS is considered as a functional food with benefits for mitigating MetS-related risks. Due to its resistance to digestion in both native and modified forms, RS is categorized into five types, known as RS1 to RS5 (19). RS1 is a formation of natural starch granules that is encapsulated within indigestible plant structures, such as the cell wall or proteins, which physically hinder the interaction between RS1 and digestive enzymes. RS1 primarily exists in whole grains and beans that are not milled thoroughly. RS2 is a type of natural starch granules in raw potatoes or green bananas, with high content of amylose, high starch density and a unique crystalline form. The property of RS2 exhibits resistance to hydrolysis by digestive enzymes to some extent. RS3, a type of retrograded starch, is produced when the starch is heated to gelatinization and then undergoes a retrogradation process at a low temperature. The gelatinization-retrogradation cycle in starch creates a crystal structure that resisted digestion by enzymes. RS4 is resistant to digestive enzymes by chemically altering the functional groups or adding new functional groups of original starch, leading to the formation of carboxymethyl starch, starch ether, starch ester, and cross-linked starch. Combination of the extended branches of amylose with fatty acids generated RS5, a starch-FA complex, where the helical structure is hardly digested by amylase. These native and modified forms can affect functionality, digestibility and fermentability in a food product (20, 21). The previous review has taken insight into the impact of properties on physiological effects and mechanisms specific to each type of RS (22). In contrast, a recent review has qualitatively discussed that RS2 and RS3 are mostly examined their cardiometabolic effects in randomized controlled trials (RCTs), highlighting the inconclusive results (23). Overall, challenges still persist in understanding the functionality of RS and in accurately reporting its effects.

Due to the research gap between the expected functionality and actual effects of RS, this systematic review and meta-analysis on the effects of RS consumption on anthropometric and serum parameters in adults with MetS-related risks was necessary. Therefore, the review aimed to qualitatively and quantitatively assess the effects of RS consumption on cardiometabolic outcomes including anthropometric parameters, serum glycemic and lipid profiles, serum inflammatory factors, and serum oxidative stress biomarkers. Although RS effects on health are well-conceived, we assumed that studies were heterogeneous in terms of type of RS, delivery mode, participant characteristics, and dose and duration of intervention, which could influence the treatment outcomes. Hence, subgroup analysis on aforementioned factors contributing to heterogeneity was explored.

## 2 Materials and methods

### 2.1 Retrieve identification

This review was conducted in accordance with the PRISMA (Preferred Reporting Items for Systematic Reviews and Meta-Analyses) guidelines. A comprehensive retrospective search was performed across five major literature databases—PubMed, Web of Science, Cochrane Library, Embase, and Scopus—covering publications up to January 21, 2025. The search strategy of five public databases was provided in Supplementary Table 1. In addition, all relevant systematic reviews containing applicable biomarkers or outcome indicators were manually screened to ensure that no eligible studies were omitted.

### 2.2 Literature screening and eligibility

Two independent investigators screened the literature and assessed study eligibility based on predefined inclusion and exclusion criteria. In cases of disagreement, a third investigator was consulted to reach a consensus. The initial screening was performed by reviewing titles and abstracts for references to treatment efficacy or biological effects. Studies were included if they met the following criteria:

1. published in English;
2. involved participants with MetS-related risks, including overweight, obesity, insulin resistance, MetS, prediabetes, T2DM, hyperlipidemia, or NAFLD;
3. employed a RCT assignment, either parallel or crossover;
4. enrolled adult participants;
5. included an intervention group receiving RS supplementation and a control group receiving either a placebo or standard starch;
6. had a minimum intervention duration of one week;
7. provided sufficient outcome data for effect size estimation, including anthropometric, glycemic, lipid, inflammatory, or oxidative stress markers.

Studies were excluded if they met any of the following criteria:

1. were non-original publications or duplicate reports;
2. used resistant dextrin instead of resistant starch as the intervention;
3. did not report the RS dosage clearly or reported the lifestyle recommendation;
4. the full text was not accessible.

### 2.3 Data extraction and characterization

Essential data were extracted from each included study, including the first author, year of publication, trial registration ID (if available), assignment model, study population, intervention details (RS type, dose and total intake), control substance characteristics (dose and total intake), intervention duration, and any reported washout period. Participant characteristics were recorded, including the total sample size, allocation to intervention and control groups, sex distribution, mean age, BW, and BMI. Effect sizes and safety outcomes were also collected. Effect sizes were categorized into five domains: (1) Anthropometric parameters, including BW, BMI, waist circumference (WC), hip circumference (HC), waist-to-hip ratio (WHR), fat mass (FM), body fat percentage, diastolic blood pressure (DBP), and systolic blood pressure (SBP); (2) Glycemic profiles, comprising fasting blood glucose (FBG), fasting insulin (FINS), glycated hemoglobin (HbA1c), homeostatic model assessment of insulin resistance (HOMA-IR), and beta-cell function (HOMA-β); (3) Lipid profiles, including TG, total cholesterol (TC), HDL-C, and low-density lipoprotein cholesterol (LDL-C); (4) Inflammatory markers, such as high-sensitivity C-reactive protein (hs-CRP), tumor necrosis factor-alpha (TNF-α), and interleukin-6 (IL-6); (5) Oxidative stress biomarkers, specifically malondialdehyde (MDA) and superoxide dismutase (SOD). Concerning their critical role in functionality, the parameters of intervention substances, including food source, purity, content analytical method, delivery mode, and feasibility, were also reported.

### 2.4 Quality assessment

Risk of bias was assessed using the Cochrane Risk of Bias (ROB) tool implemented in Review Manager version 5.3. The tool includes seven domains: (1) random sequence generation, (2) allocation concealment, (3) blinding of participants and personnel, (4) blinding of outcome assessment, (5) incomplete outcome data, (6) selective reporting, and (7) other sources of bias. The overall ROB judgment of each study was provided based on the previous domains. The Jadad scale was used to evaluate methodological quality. The scale awards up to five points based on the following criteria: (1) randomization (1 point for stating randomization, plus 1 additional point for appropriate randomization methods); (2) blinding (1 point for stating double-blinding, plus 1 additional point for appropriate blinding methods); and (3) description of withdrawals and dropouts (1 point for reporting the number and reasons for withdrawals).

### 2.5 Statistical analysis

Effect sizes were calculated by comparing the mean net changes between the intervention and control groups. The standard deviation (SD) of the net change was estimated using the following formula:


$$\mathrm{SD}=\sqrt{\left[\begin{array}{l} SD_{\mathrm{pre}-\text{treatment}}^{2}+SD_{\text{post}-\text{treatment}}^{2}-\\ \left(2\times R\times SD_{\mathrm{pre}-\text{treatment}}\times SD_{\text{post}-\text{treatment}}\right) \end{array}\right]}.$$

A correction coefficient (*R*) of 0.5 was assumed for the calculation of standard deviations. For studies that presented data using the median and interquartile range, established mathematical methods were employed to transform these values into the mean and SD (24, 25). If standard error (SE) was reported in some studies, the formula between SD and SE was used to convert to SD using the following formula:


$$\mathrm{SD}=\mathrm{SE}\times \sqrt{n.}$$

Here, *n* meant the number of participants in the group. For studies presenting effect sizes in bar charts, mean values and SDs were extracted using ImageJ software. When outcomes were reported separately for subgroups (e.g., males and females), combined means and SDs were calculated using an online statistical tool.^1^ Each effect size was expressed as mean difference (MD) with corresponding 95% confidence intervals (CI). In cases where units could not be standardized across studies or when there were substantial differences in measurement scales, standard mean difference (SMD) with 95% CI were calculated. A random-effects model was employed for all meta-analyses to account for inter-study variability. Statistical heterogeneity was assessed using the *I*^2^ statistic, with values <50% considered low heterogeneity and ≥50% considered high. To explore potential sources of heterogeneity, subgroup analysis was conducted based on RS type (RS2 or RS3), delivery mode (supplement or food), dose (<30 g/day or ≥30 g/day) and duration (<8 weeks or ≥8 weeks) of intervention, geographic region (western developed countries or others), disease type (overweight/obesity, MetS, insulin resistance, prediabetes/T2DM, hyperlipidemia, or NAFLD), mean age (<45 years or ≥45 years), mean BMI (<30 kg/m^2^ or ≥30 kg/m^2^), overall ROB judgment (low, unclear or high), the score of the Jadad scale (low score of no more than 3 or high score of more than 3) and duration (<8 weeks or ≥8 weeks) of intervention, and assignment (crossover or parallel). Subgroup differences were assessed using Chi-square tests following subgroup meta-analysis. Sensitivity analysis was conducted within studies of non-high ROB judgment to investigate robustness of results. All meta-analyses were performed using Review Manager. Publication bias was assessed visually via funnel plots and statistically using Begg’s and Egger’s tests. If Egger’s test yielded a significant *p* value for a specific effect size, the Trim and Fill method was applied to estimate adjusted results. Publication bias was performed using Stata version 15.1. A *p* value < 0.05 was considered statistically significant in the present review.

## 3 Results

### 3.1 Search results

**Figure 1:**
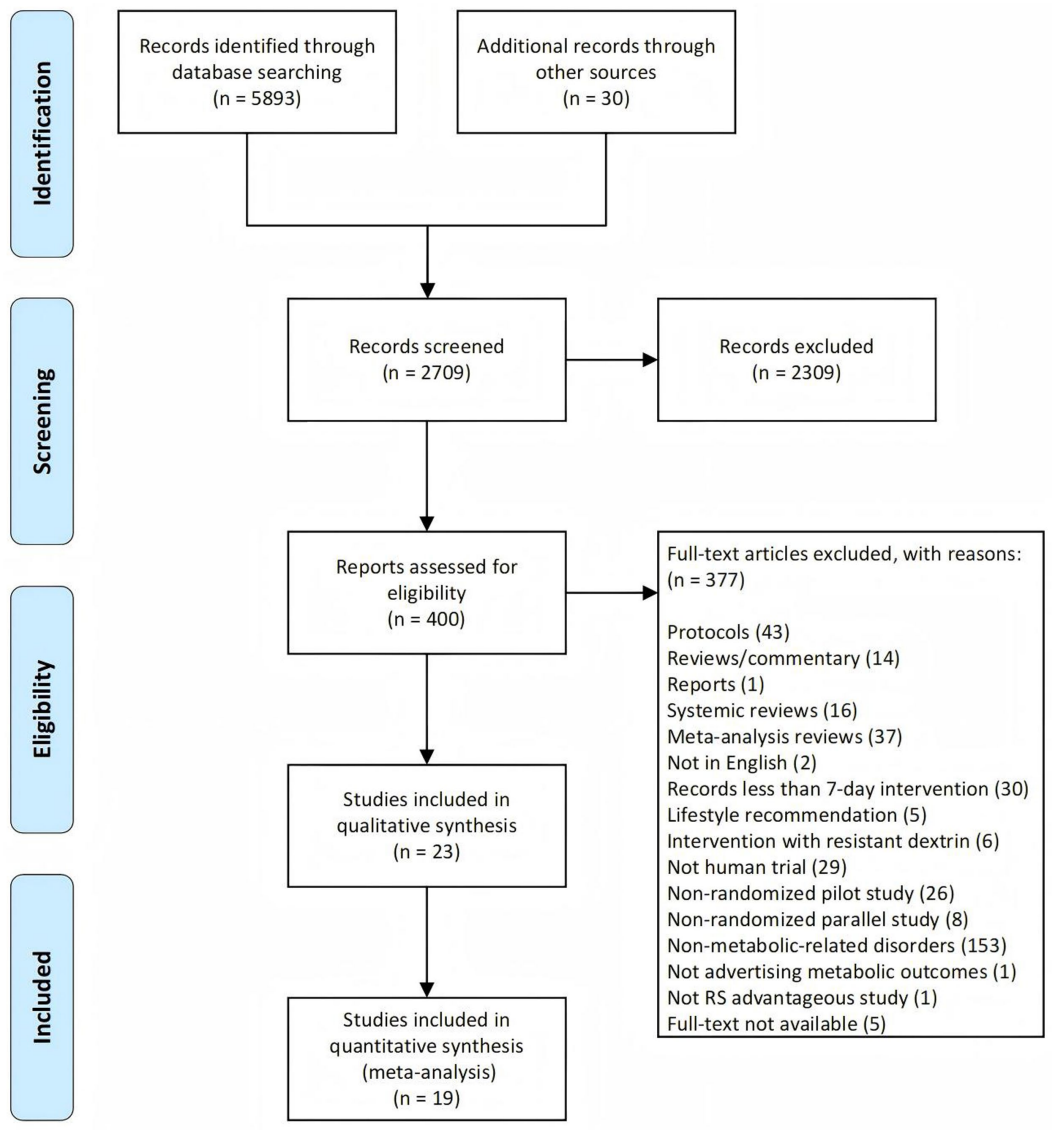
PRISMA flowchart illustrating the study selection process. PRISMA, Preferred Reporting Items for Systematic Reviews and Meta-Analyses.

The study selection process was illustrated in the PRISMA flow diagram (Figure 1). A total of 5,893 records were identified through searches of public literature databases, and an additional 30 records were retrieved from previously published systematic reviews. After removing duplicates, 2,709 unique records remained. Two reviewers (RD and RZ) independently screened the records, and disagreements were resolved by a third reviewer (XM). Of the 2,709 records, 400 articles were deemed potentially eligible based on title and abstract screening. After full-text review, 377 articles were excluded for reasons detailed in Figure 1, resulting in 23 studies included for qualitative synthesis. Among these, 19 studies with extractable effect size data were further included in the quantitative synthesis (meta-analysis).

### 3.2 Study characteristics

Table 1 summarized the characteristics of the 23 studies included in this review, published over a 20-year period from 2004 to 2024. Of these, 11 were crossover randomized controlled trials, and 12 were parallel-group trials. Geographically, 11 studies were conducted in Western developed countries: one in Canada (26), one in Denmark (27), one in France (28), three in the United Kingdom (15, 29, 30), and five in the United States (31–35). The remaining 12 studies were conducted in other regions: one in Brazil (36), four in China (10, 11, 37, 38), one in Indonesia (39), three in Iran (40–42), two in South Korea (43, 44), and one in Mexico (45). The included studies covered five categories of metabolic conditions: seven focused on overweight or obesity, six on MetS, eight on T2DM, one on hyperlipidemia, and one on NAFLD. RS doses ranged from 6 to 40 g/day, with intervention durations varying from 4 to 16 weeks. In Maki’s study, the data from higher-dose and lower-dose groups were reported separately; therefore, these two groups were considered as distinct trials in the subsequent analysis. The matching criteria for RS and control interventions were categorized as follows: 12 studies used dose-matched controls, four used carbohydrate-matched controls, five used energy-matched controls, two were unmatched, and one lacked matching details.

**Table 1:** Study characteristics of included studies.

Study, Author/Year	Country	Registration ID	Study design	Subjects	Resistant starch type	Intervention (dose, g/day)	Control (dose, g/day)	Follow-up, weeks	Washout, weeks	Matching type
Ble-Castillo 2010 (45)	Mexico	–	Randomized, single-blind, crossover trial	Obesity with T2DM	RS2	Native banana starch (8.16/24)	Soy milk (0/24)	4	0	Dose match
Bodinham 2012 (29)	UK	–	Randomized, single-blind, crossover trial	Overweight with insulin resistance	RS2	Hi-maize 260 (40/67)	Amioca (0/27)	12	4	Carbohydrate match
Bodinham 2014 (30)	UK	–	Randomized, single-blind, crossover trial	T2DM	RS2	Hi-maize 260 (40/67)	Amioca (0/27)	12	12	Carbohydrate match
Cao 2022 (31)	USA	NCT03624569	Randomized, open-label, crossover trial	MetS	RS3	Retrograded potato (17.5/350)	Bagel (0/100)	2	2	Energy match
Costa 2019 (36)	Brazil	NCT03230123	Randomized, open-label, parallel trial	Overweight with prediabetes or T2DM	RS2	Native banana biomass (4.5/40)	None (0/0)	24	Not need	Not match
Dainty 2016 (26)	Canada	NCT02129946	Randomized, double-blind, crossover trial	Overweight or obesity	RS2	Bagel containing Hi-maize 260 (25/119.8)	Control Bagel (6.83/124.2)	8	4	Energy match
Eshghi 2019 (40)	Iran	NCT01992783	Randomized, double-blind, crossover trial	Overweight or obesity	RS2	Hi-maize 260 (8.1/13.5)	Maltodextrin (0/13.5)	12	4	Dose match
Gargari 2015 (41)	Iran	IRCT201110293253N4	Randomized, triple-blind, parallel trial	T2DM	RS2	Hi–maize 260 (6/10)	Maltodextrin (0/10)	8	Not need	Dose match
Johnston 2010 (15)	UK	ISRCTN35312139	Randomized, single-blind, parallel trial	Overweight with insulin resistance	RS2	Hi-maize 260 (40/67)	Amioca (0/27)	12	Not need	Carbohydrate match
Karimi 2016 (42)	Iran	IRCT201110293253N4	Randomized, triple-blind, parallel trial	Overweight with T2DM	RS2	Hi-maize 260 (6/10)	Maltodextrin (0/10)	8	Not need	Dose match
Kwak 2012 (43)	South Korea	–	Randomized, double-blind, parallel trial	T2DM	RS2	Rice containing maize RS (6.5/210)	Control rice (0/210)	4	Not need	Dose match
Li 2024 (12)	China	ChiCTR-TTRCC-13003333	Randomized, double-blind, crossover trial	Overweight or obesity	RS2	Hi-maize 260 (40/91.2)	Amioca (0/72)	8	4	Energy match
Maki 2012 (32)	USA	NCT01058135	Randomized, double-blind, crossover trial	Overweight or obesity	RS2	Hi-maize 260 (18/30)	Amioca (0/11.6)	4	3	Not match
Maki 2012 (32)	USA	NCT01058135	Randomized, double-blind, crossover trial	Overweight or obesity	RS2	Hi-maize 260 (9/15)	Amioca (0/11.6)	4	3	Energy match
Maziarz 2017 (33)	USA	–	Randomized, double-blind, parallel trial	Overweight or obesity	RS2	Muffins containing Hi-maize 260 (30/180)	Control muffins (0/180)	6	Not need	Dose match
Meng 2019 (38)	China	–	Randomized, open–label, parallel trial	Early type diabetic nephropathy	RS2	High RS, low–protein flour (17.41/50)	Common staple (0/50)	12	Not need	Dose match
Miao 2024 (10)	China	ChiCTR2200062871	Randomized, double-blind, parallel trial	Hyperlipidemia	RS3	*Canna edulis* resistant starch (9.6/20)	*Canna edulis* native starch (1.2/20)	12	Not need	Dose match
Ni 2023 (11)	China	ChiCTR-IOR-15007519	Randomized, double-blind, parallel trial	NAFLD	RS2	Hi–maize 260 (40/91.2)	Amioca (0/72)	16	Not need	Energy match
Park 2004 (44)	South Korea	–	Randomized, double-blind, parallel trial	Overweight	RS3	Retrograded maize RS (24/40)	Maize starch (0/40)	3	Not need	Dose match
Penn-Marshall 2010 (34)	USA	–	Randomized, double-blind, crossover trial	Overweight with T2DM	RS2	Bread containing Hi–maize 260 (12.4/121.8)	Control bread (3.2/121.8)	6	2	Dose match
Peterson 2018 (35)	USA	NCT01708694	Randomized, double-blind, parallel trial	Obesity with prediabetes	RS2	Hi–maize 260 (27/45)	Amioca (0/45)	12	Not need	Dose match
Robertson 2012 (28)	France	ISRCTN56997186	Randomized, single-blind, crossover trial	Obesity with insulin resistance	RS2	Hi–maize 260 (40/67)	Amioca (0/27)	8	8	Carbohydrate match
Schioldan 2018 (27)	Denmark	NCT01584427	Randomized, double-blind, crossover trial	MetS	RS2	Healthy carbohydrate diet containing RS (21/534)	Western–style diet (3/541)	4	4–6	Unknown
Sunarti 2022 (39)	Indonesia	–	Randomized, single-blind, parallel trial	Overweight or obesity	RS2	Test snacks (4.0/42)	Standard snacks (1.5/42)	6	Not need	Dose match

MetS, metabolic syndrome; NAFLD, non-alcohol fatty liver disease; RS, resistant starch; T2DM, type 2 diabetes mellitus.

A total of 1,073 participants were included across the studies, comprising 430 males and 545 females, with sex-specific data unavailable in two trials. The participants were aged between 31 and 66.1 years, with body weight ranging from 71.8 to 116.1 kg and BMI values between 24.5 and 37.7 kg/m^2^. Effect sizes and safety outcomes are summarized in Table 2. Notably, two studies were identified as having the same registration ID (41, 42). Consequently, to avoid duplication, only one effect size from each study was selected for analysis.

**Table 2:** Study characteristics on subjects’ information.

Study, Author/Year	Size, I/C	Sex, Male/Female	Age, years (I vs. C)	Weight, kg (I vs. C)	BMI, kg/m^2^ (I vs. C)	Outcomes	Safety report
Ble-Castillo 2010 (45)	28, 28/28	28, 4/24	51.70 ± 5.60	79.00 ± 16.63	34.89 ± 2.32	BW, BMI, waist-to-hip ratio, body fat percentage, TG, TC, HDL-C, FBG, FINS, HbA1c, HOMA-IR	Not provided
Bodinham 2012 (29)	12, 12/12	12, 8/4	37.0 ± 4.0	–	28.2 ± 0.4	BW, BMI, WC, FM, body fat percentage, SBP, DBP, FBG, FINS, TG, TC	Not reported
Bodinham 2014 (30)	17, 17/17	17, 12/5	55 ± 2.4	–	30.6 ± 1.3	BW, BMI, FM, HbA1c, FBG, FINS, HbA1c, HOMA-β, TG, TC, HDL-C, LDL-C	Mild flatulence
Cao 2022 (31)	27, 27/27	27, 13/14	32.5 ± 1.3	–	35.0 ± 1.0	BMI, WC, SBP, DBP, FBG, FINS, HOMA-IR, MDA	Not reported
Costa 2019 (36)	113, 62/51	62, 17/45	66.1 ± 6.8	77 ± 16	30.0 ± 5.2	BW, BMI, WC, HC, waist-to-hip ratio, SBP, DBP, FBG, FINS, HbA1c, HOMA-IR, TG, TC, HDL-C, LDL-C,	Not reported
		51, 9/42	65.1 ± 8.4	75 ± 14	29.5 ± 5.0		
Dainty 2016 (26)	24, 24/24	24, 16/8	55.30 ± 1.59	90.40 ± 2.25	30.2 ± 0.57	FBG, FINS, HOMA-IR, HOMA-β	Not provided
Eshghi 2019 (40)	21, 21/21	21, 13/8	35 ± 7.0	90.5 ± 9.8	32.5 ± 3.5	BW, BMI, WC, SBP, DBP, FBG, FINS, HOMA-IR, TG, TC, HDL-C, LDL-C, SOD, MDA	Not provided
Gargari 2015 (41)	60, 28/32	28, 0/28	49.5 ± 8.0	74.2 ± 4.3	31.5 ± 4.5	TG, TC, HDL-C, LDL-C, hs-CRP, TNF-α, IL-6	Not provided
		32, 0/32	49.6 ± 8.4	71.8 ± 3.5	30.8 ± 5.2		
Johnston 2010 (15)	20, 10/10	10, un/un	45.20 ± 3.55	–	31.3 ± 1.70	BMI, HOMA-IS, HOMA-β	Not provided
		10, un/un	50.10 ± 4.05	–	30.4 ± 1.15		
Karimi 2016 (42)	56, 28/28	28, 0/28	49.5 ± 8.0	74.2 ± 4.3	31.5 ± 4.5	FBG, FINS, HbA1c, HOMA-IR, hs-CRP, SOD, MDA	Not reported
		28, 0/28	48.6 ± 7.9	73.9 ± 5.5	31.0 ± 4.9		
Kwak 2012 (43)	85, 41/44	41, 21/20	51.7 ± 2.03	–	25.0 ± 0.49	FBG, FINS, HOMA-IR, SOD, MDA	Not reported
		44, 26/18	49.4 ± 1.74	–	24.5 ± 0.37		
Li 2024 (12)	37, 37/37	37, 22/15	33.43 ± 7.71	83.24 ± 80.91	27.93 ± 3.75	BW, BMI, WC, HC, waist-to-hip ratio, FM, body fat percentage, SBP, DBP, TG, TC, HDL-C, LDL-C, TNF-α, IL-6	Not reported
		37, 22/15	33.43 ± 7.71	82.74 ± 15.67	28.58 ± 3.79		
Maki 2012 (32)	33, 33/32/33	33, 11/22	49.5 ± 1.6	–	30.6 ± 0.5	FBG	1 constipation
		32, 11/21	48.1 ± 3.3	–	30.7 ± 0.9		
		33, 11/22	50.2 ± 1.7	–	30.6 ± 0.5		
Maziarz 2017 (33)	18, 11/7	11, 2/9	31.0 ± 3.0	–	34.8 ± 1.5	FBG, FINS	Not reported
		7, 1/6	31.2 ± 4.2	–	30.6 ± 1.5		
Meng 2019 (38)	70, 34/36	34, 18/16	62.85 ± 9.3	–	26.4 ± 3.9	FBG, HbA1c, TG, TC, HDL-C, LDL-C, TNF-α, IL-6, SOD, MDA	One sample reported
		26, 21/15	61 ± 9.5	–	25.8 ± 3.6		
Miao 2024 (10)	73, 38/35	73, 24/49	48.3 ± 10.2	–	25.5 ± 3.6	TG, TC, HDL-C, LDL-C, SOD, MDA	Not provided
		73, 24/49	52.0 ± 9.2	–	24.3 ± 2.7		
Ni 2023 (11)	196, 99/97	99, 73/26	39.20 ± 1.70	83.52 ± 2.86	28.31 ± 0.76	BW, BMI, WC, HC, waist-to-hip ratio, FM, body fat percentage, SBP, DBP, FBG, FINS, HOMA-IR, TG, TC, HDL-C, LDL-C, TNF-α, IL-6	No serious adverse events, 8 constipations, 20 flatulence, and 35 intestinal exhausts
		97, 69/28	38.91 ± 1.91	84.24 ± 3.03	28.74 ± 0.78		
Park 2004 (44)	25, 12/13	12, 0/12	42.3 ± 3,1	65.0 ± 2.0	26.6 ± 0.7	BW, BMI, body fat percentage, WC, HC, SBP, DBP, FBG, FINS, TG, TC, LDL-C, HDL-C	Not provided
		13, 0/13	43.6 ± 2.8	68.6 ± 1.6	27.9 ± 0.5		
Penn-Marshall 2010 (34)	15, 15/15	15, 8/7	36.6 ± 1.6	116.1 ± 8.2	37.7 ± 2.0	FBG, FINS, HOMA-IR, HOMA-β	Not provided
Peterson 2018 (35)	59, 29/30	29, 15/14	55 ± 10	98.1 ± 14.6	35.7 ± 5.2	BW, FM, SBP, DBP, FBG, FINS, HbA1c, TG, TC, HDL-C, LDL-C, hs-CRP, TNF-α	23 reports
		30, 5/25	54 ± 10	103.3 ± 13.3	35.5 ± 4.4		
Robertson 2012 (28)	15, 15/15	15, 8/7	48.9 ± 3.9	99.1 ± 7.3	33.8 ± 1.9	BW, BMI, FM, FFM, SBP, FBG, FINS, HOMA-IR, HOMA-β, TG, TC	Not provided
Schioldan 2018 (27)	19, 19/19	19, 14/5	–	100.5 ± 19.8	–	BW, SBP, DBP, FBG, FINS, HOMA-IR, TG, TC, HDL-C, LDL-C	Not provided
		19, 14/5	–	98.7 ± 17.2	–		
Sunarti 2022 (39)	50, 25/25	25, un/un	54.3 ± 9.5	78.6 ± 18.6	30.7 ± 5.0	BW, BMI, body fat percentage, TG, TC, HDL-C, LDL-C, TNF-α	Not provided
		25, un/un	52.9 ± 10.3	76.3 ± 15.7	30.4 ± 4.7		

C, control group; I, intervention group; BW, body weight; BMI, body mass index; WC, waist circumference; HC, hip circumference; WHR, waist-to-hip ratio; FM, fat mass; DBP, diastolic blood pressure; SBP, systolic blood pressure; FBG, fasting blood glucose; FINS, fasting insulin; HbA1c, glycated hemoglobin; HOMA-IR, homeostatic model assessment of insulin resistance; HOMA-β, homeostatic model assessment of beta-cell function; TG, triglycerides; TC, total cholesterol; HDL-C, high-density lipoprotein cholesterol; LDL-C, low-density lipoprotein cholesterol; hs-CRP, high-sensitivity C-reactive protein; TNF-α, tumor necrosis factor-alpha; IL-6, interleukin-6; MDA, malondialdehyde; SOD, superoxide dismutase.

The properties of included studies were documented in Supplementary Table 2. Among these, the most of 20 studies using RS2, and remaining three using RS3. In terms of the sources of RS, 16 studies identified maize as the primary source, two studies focused on banana, one study on potato, one on canna edulis, one on both maize and potato, and one study utilized multiple sources. RS substances had a purity of 34–34.8% when delivered as a powdered supplement in 15 studies, and 3.1–21.2% (26) when delivered as food matrices, including diet, rice, bread, bagel, and snack, in eight studies. The studies utilized Hi-maize 260, whose purity was confirmed by AOAC method 991.43. Besides, two studies employed AOAC 2002.02, two studies utilized the Goni method, one study used a total dietary fiber determination kit, and the remaining three studies did not specify the method used.

### 3.3 Data synthesis and quality assessment

Four studies were excluded from the meta-analysis due to the absence of net changes of effect sizes. In Maki’s study, effect sizes for the higher-dose and lower-dose groups were calculated separately. Each effect size included in the meta-analysis was derived from a minimum of three studies. Most of effect sizes were pooled as MDs, while some effect sizes, such as HOMA-β, hs-CRP, TNF-α, SOD, and MDA, were pooled as SMDs.

**Figure 2:**
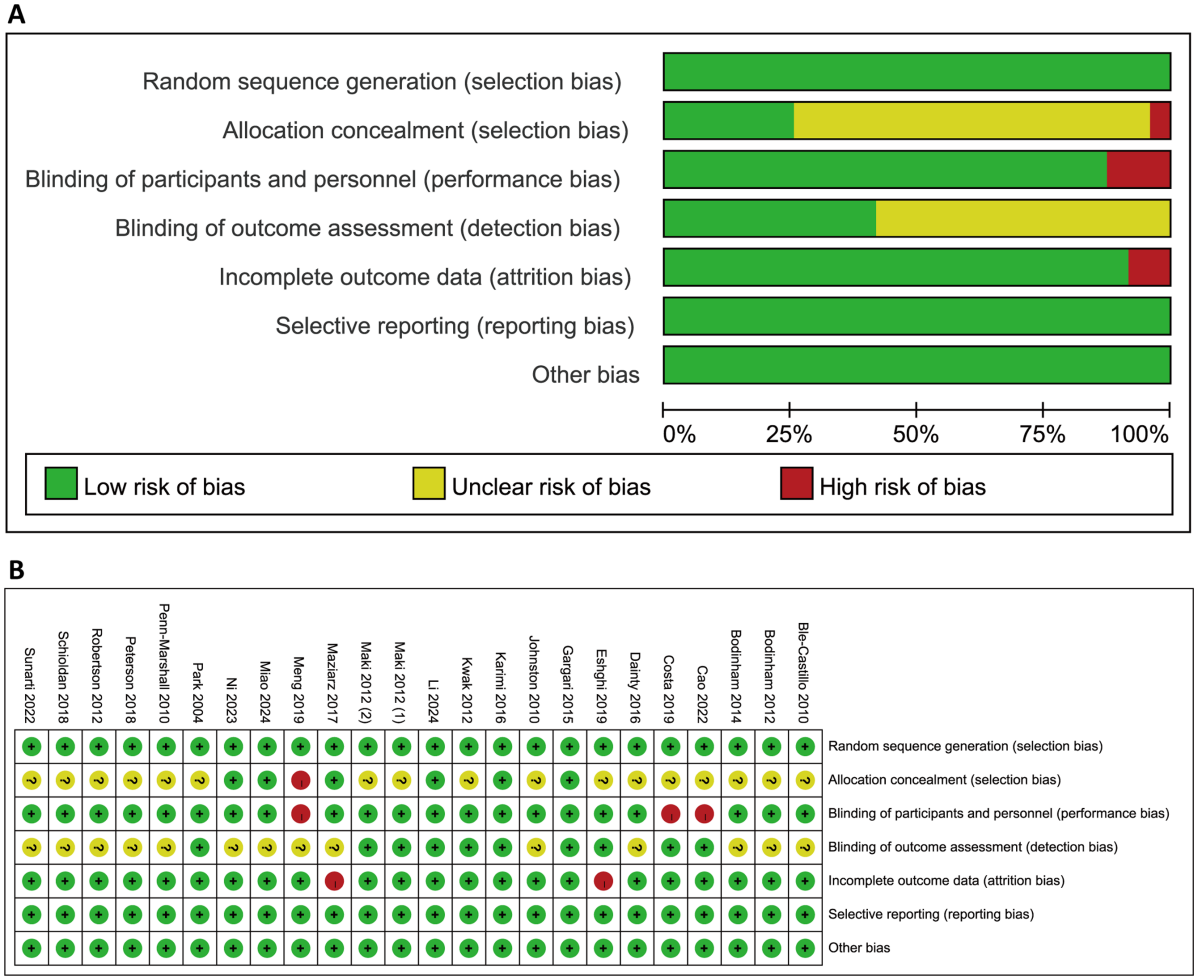
Quality assessment for the included studies using Cochrane risk of bias tool. **(A)** Proportions of studies exhibiting different levels of bias across the seven assessed domains. **(B)** Risk of bias judgment for each study. Symbols indicated the use of bias-reducing measures: “+” denotes low risk (i.e., bias-minimizing method used), “−” denotes high risk (i.e., method not used), and “?” indicates unclear risk (i.e., insufficient information).

The results of quality assessment using Cochrane ROB tool were presented in Figure 2 and Supplementary Table 3. All included studies were judged as low risk for random sequence generation. Allocation concealment was assessed as low risk in five studies, while one study was judged as high due to lack of concealment; the remaining 16 studies did not report this information. Blinding of participants and personnel was considered adequate in 20 studies, with three studies rated as high risk in this domain. Regarding blinding of outcome assessment, eight studies were rated as low risk, while 15 studies did not provide sufficient details. For incomplete outcome data, 21 studies demonstrated as low risk, whereas two studies were rated as high due to dropout rates exceeding 20%. Selective reporting and other potential sources of bias were also assessed, and all 23 studies were considered low risk in both categories. For overall ROB judgment, three studies were judged as low risk, 15 were as unclear, and five were as high. The results of the Jadad scale assessments are provided in Supplementary Table 4. In brief, seven studies scored 2 points, two studies scored 3 points, 11 studies scored 4 points, and four studies achieved the maximum score of 5.

### 3.4 Anthropometric parameters

**Figure 3:**
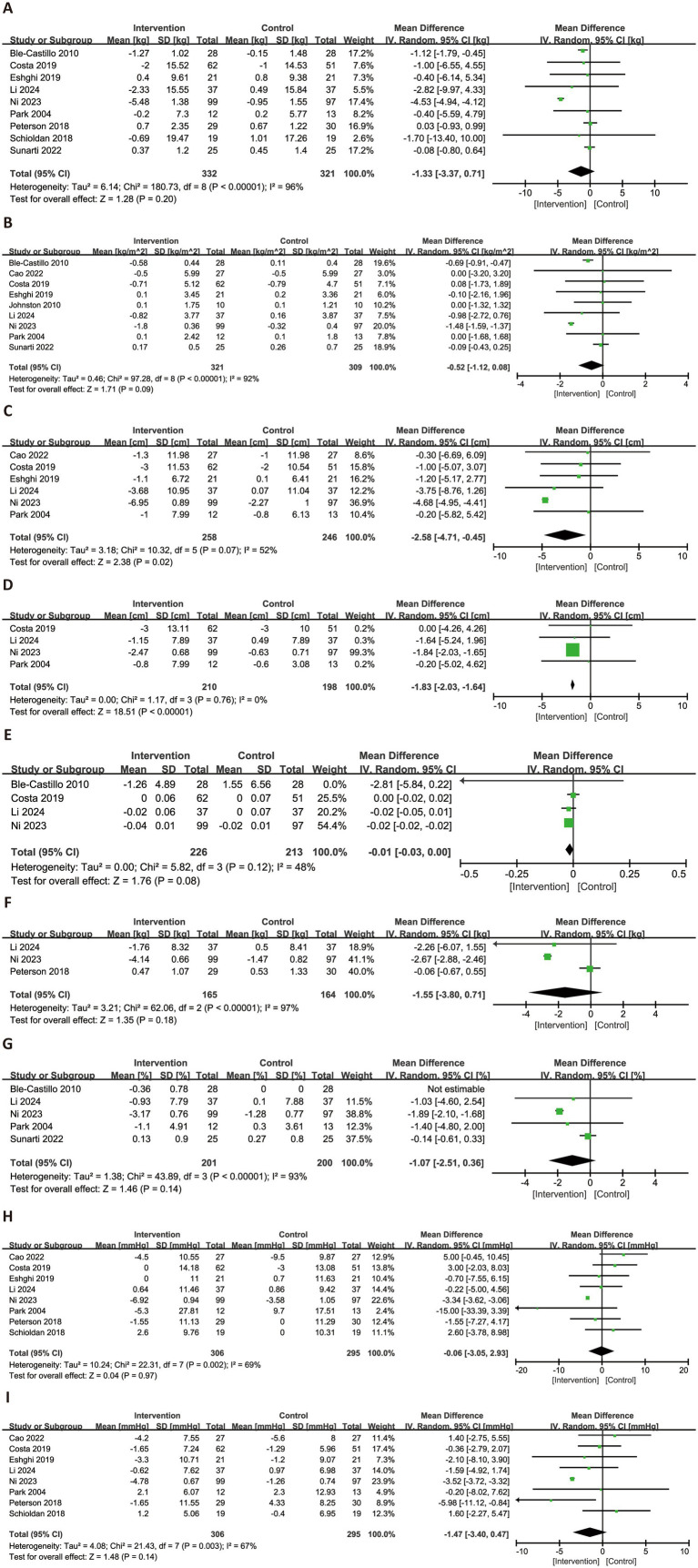
Forest plots illustrating the effects of RS versus control on anthropometric parameters. Outcomes included: BW **(A)**, BMI **(B)**, WC **(C)**, HC **(D)**, waist-to-hip ratio **(E)**, FM **(F)**, body fat percentage **(G)**, SBP **(H)**, and DBP **(I)**. BW, body weight; BMI, body mass index; WC, waist circumference; HC, hip circumference; WHR, waist-to-hip ratio; FM, fat mass; DBP, diastolic blood pressure; SBP, systolic blood pressure.

Nine anthropometric outcomes were evaluated across the included studies (Figure 3). Nine trials assessed the effects of RS consumption on BW and BMI, with no statistically significant differences observed (BW: MD = −1.33 kg, 95% CI: −3.37 to 0.71, *p* = 0.20, *I*^2^ = 96%; BMI: MD = −0.52 kg/m^2^, 95% CI: −1.12 to 0.08, *p* = 0.09, *I*^2^ = 92%). In terms of body shape, RS intake was associated with a significant reduction in WC (MD = −2.58 cm, 95% CI: −4.71 to −0.45, *p* = 0.02, *I*^2^ = 52%; six trials) and HC (MD = −1.83 cm, 95% CI: −2.03 to −1.64, *p* < 0.0001, *I*^2^ = 0%; four trials). The change in waist-to-hip ratio, based on four trials, was minimal and not statistically significant (MD = −0.01, 95% CI: −0.03 to 0.00, *p* = 0.08, *I*^2^ = 48%). Regarding fat-related outcomes, RS consumption did not significantly affect FM (MD = −1.55 kg, 95% CI: −3.80 to 0.71, *p* = 0.18, *I*^2^ = 97%) or body fat percentage (MD = −1.07, 95% CI: −2.51 to 0.36, *p* = 0.14, *I*^2^ = 93%). For blood pressure parameters, no significant effects were observed. SBP showed negligible change (MD = −0.06 mmHg, 95% CI: −3.05 to 2.93, *p* = 0.97, *I*^2^ = 69%), while DBP exhibited a non-significant trend toward reduction (MD = −1.47 mmHg, 95% CI: −3.40 to 0.47, *p* = 0.14, *I*^2^ = 67%).

### 3.5 Glycemic profiles

**Figure 4:**
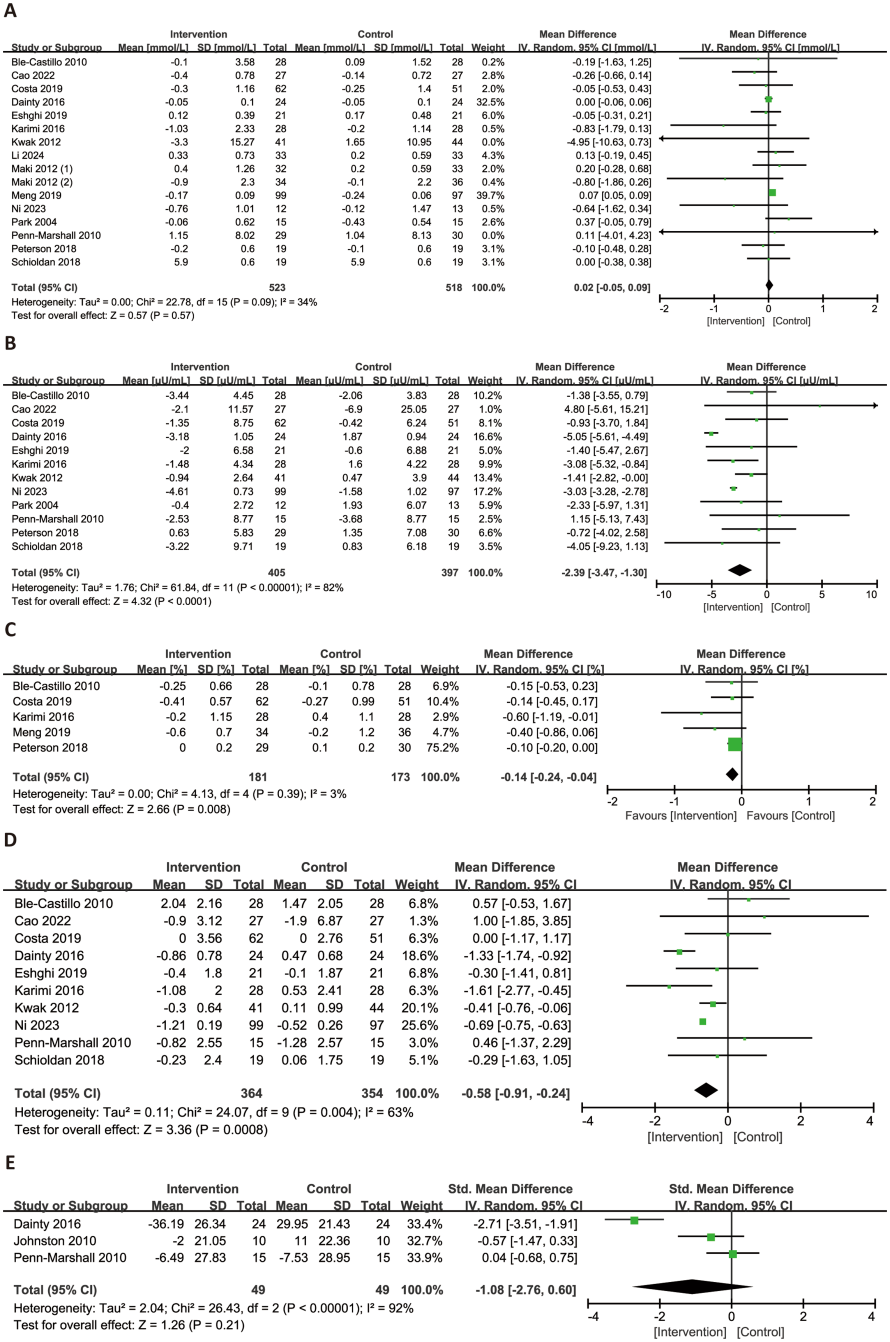
Forest plots illustrating the effects of RS versus control on glycemic profiles. Outcomes included: FBG **(A)**, FINS **(B)**, HbA1c **(C)**, HOMA-IR **(D)**, and HOMA-β **(E)**. FBG, fasting blood glucose; FINS, fasting insulin; HbA1c, glycated hemoglobin; HOMA-IR, homeostatic model assessment of insulin resistance; HOMA-β, homeostatic model assessment of beta-cell function.

Figure 4 illustrated the effects of RS consumption on glycemic profiles. A total of 16 trials evaluated FBG, with no statistically significant effect observed (MD = 0.02 mmol/L, 95% CI: −0.05 to 0.09, *p* = 0.57), and low heterogeneity (*I*^2^ = 34%). A meta-analysis of 12 trials demonstrated a significant reduction in FINS levels (MD = −2.39 μU/mL, 95% CI: −3.47 to −1.30, p < 0.0001), although substantial heterogeneity was present (*I*^2^ = 82%). For HbA1c, a significant reduction was observed with low heterogeneity (MD = −0.14, 95% CI: −0.24 to −0.04, *p* = 0.008, *I*^2^ = 3%). HOMA-IR, based on 10 trials, also showed a significant improvement (MD = −0.58, 95% CI: −0.91 to −0.24, *p* = 0.0008), though heterogeneity was high (*I*^2^ = 63%). In contrast, three trials reporting on HOMA-β were included in a separate meta-analysis, which showed a non-significant reduction (SMD = −1.08, 95% CI: −2.76 to 0.60, *p* = 0.21) and high heterogeneity (*I*^2^ = 92%).

### 3.6 Lipid profiles

**Figure 5:**
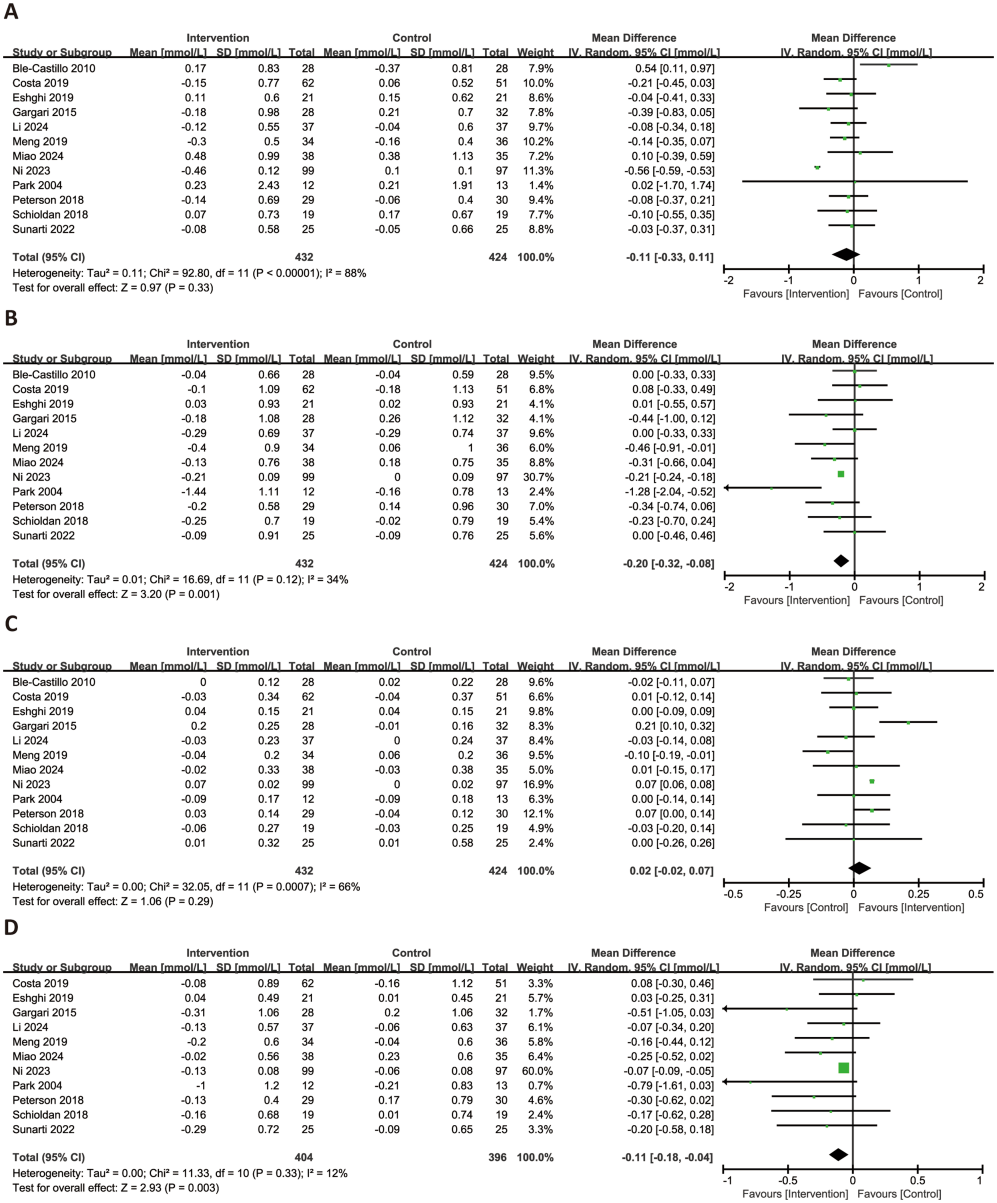
Forest plots illustrating the effects of RS versus control on lipid profiles. Outcomes included: TG **(A)**, TC **(B)**, HDL-C **(C)**, and LDL-C **(D)**. TG, triglycerides; TC, total cholesterol; HDL-C, high-density lipoprotein cholesterol; LDL-C, low-density lipoprotein cholesterol.

Figure 5 presented the forest plots for the effects of RS consumption on four lipid profile parameters. Across 12 trials, RS intake was not associated with a significant reduction in TG (MD = −0.11 mmol/L, 95% CI: −0.33 to 0.11, *p* = 0.33), with considerable heterogeneity observed (*I*^2^ = 88%). However, RS supplementation significantly reduced TC (MD = −0.20 mmol/L, 95% CI: −0.32 to −0.08, *p* = 0.001, *I*^2^ = 34%) and LDL-C (MD = −0.11 mmol/L, 95% CI: −0.18 to −0.04, *p* = 0.003, *I*^2^ = 12%), both with low heterogeneity. HDL-C levels were not significantly affected by RS intake (MD = −0.02 mmol/L, 95% CI: −0.02 to 0.07, *p* = 0.29), and substantial heterogeneity was noted (*I*^2^ = 66%).

### 3.7 Inflammatory factors

**Figure 6:**
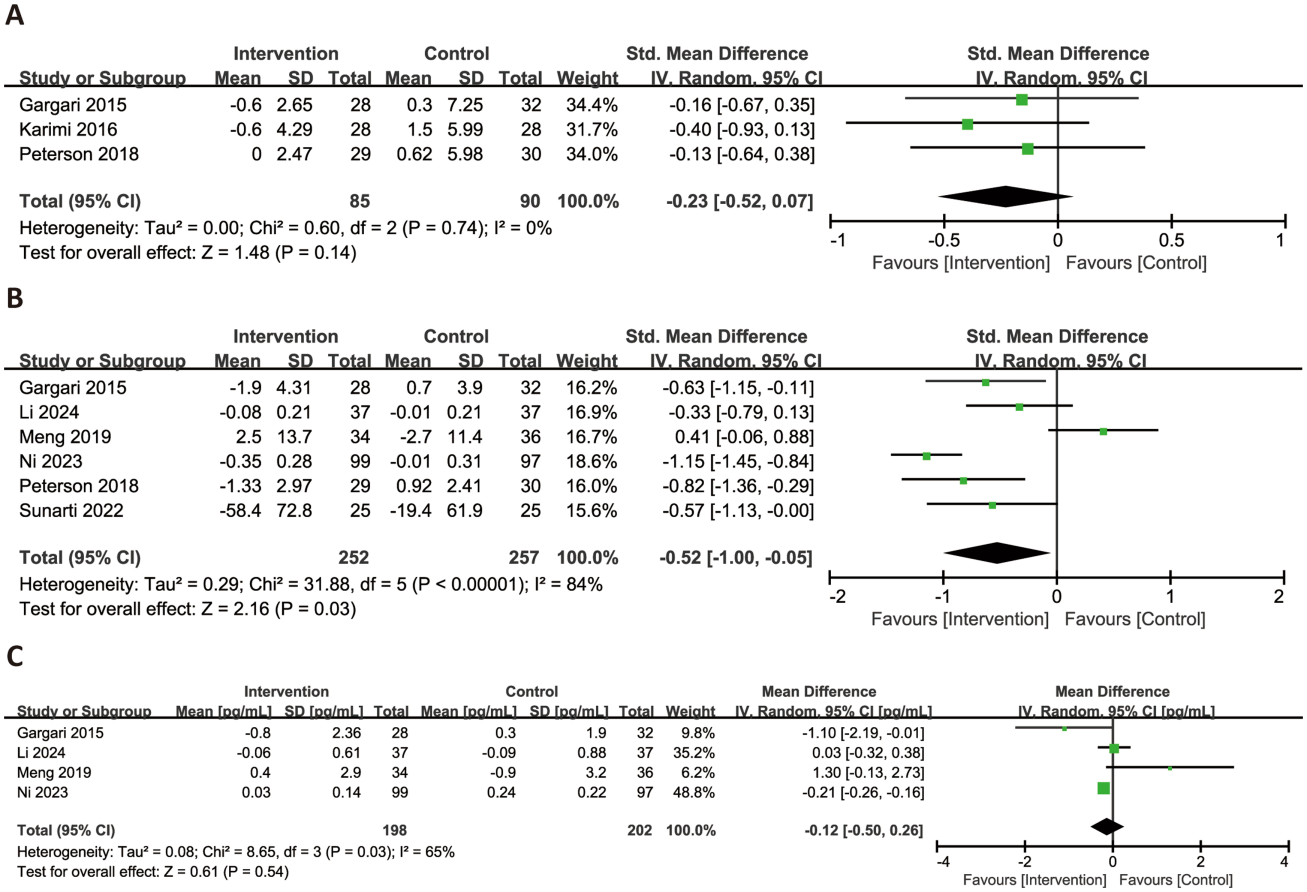
Forest plots illustrating the effects of RS versus control on inflammatory factors. Outcomes included: hs-CRP **(A)**, TNF-α **(B)**, and IL-6 **(C)**. hs-CRP, high-sensitivity C-reactive protein; TNF-α, tumor necrosis factor-alpha; IL-6, interleukin-6.

Figure 6 illustrated the effects of RS consumption on three commonly reported inflammatory markers. Based on three trials, RS intake did not significantly reduce levels of hs-CRP (SMD = −0.23, 95% CI: −0.52 to 0.07, *p* = 0.14), with no observed heterogeneity (*I*^2^ = 0%). For TNF-α, a significant reduction was observed across six trials (SMD = −0.52, 95% CI: −1.00 to −0.05, *p* = 0.03), although heterogeneity was substantial (*I*^2^ = 84%). Regarding IL-6, four trials were synthesized, and the pooled analysis revealed no significant effect (MD = −0.12 pg./mL, 95% CI: −0.50 to 0.26, *p* = 0.54), with high heterogeneity (*I*^2^ = 65%).

### 3.8 Oxidative stress biomarkers

**Figure 7:**
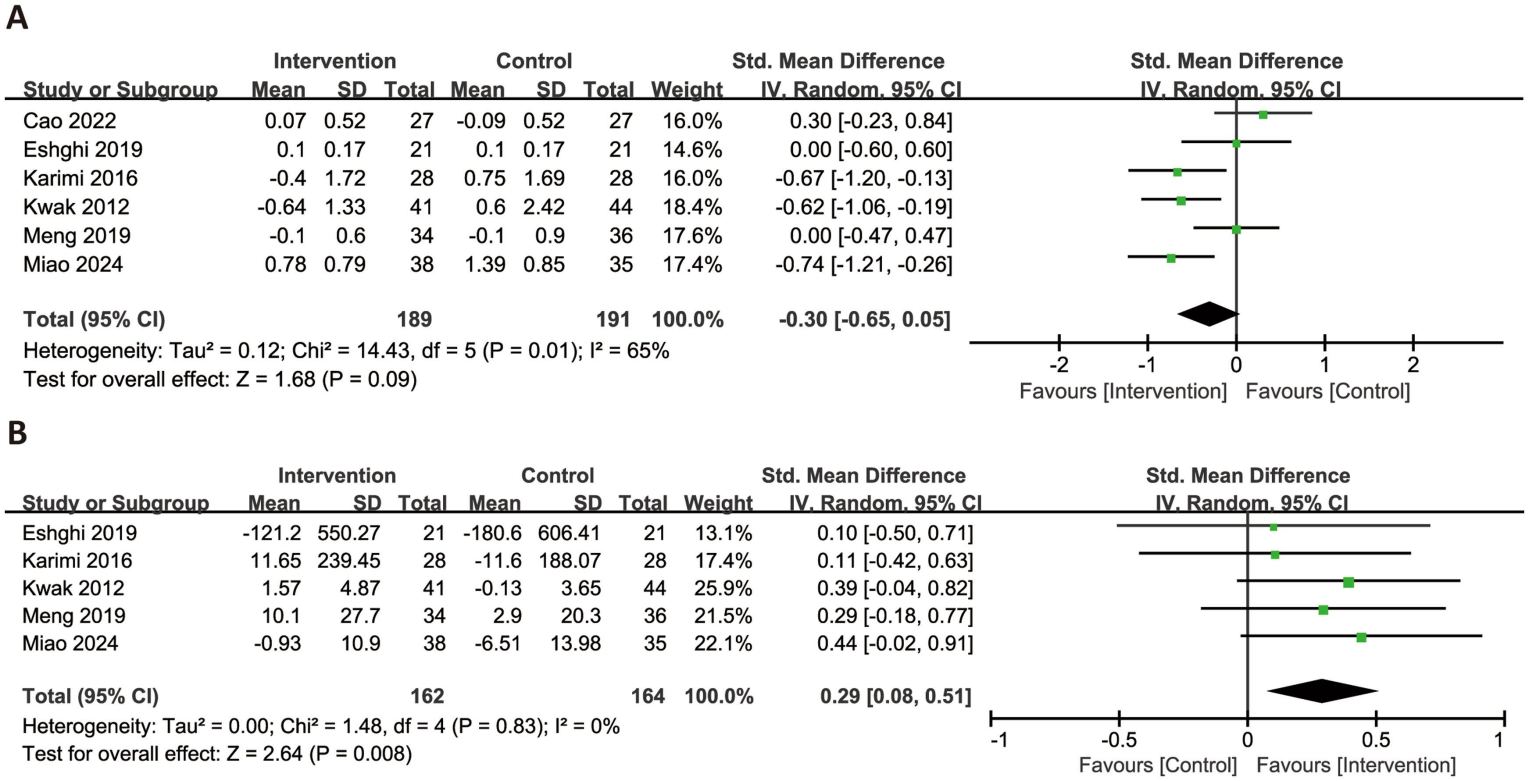
Forest plots illustrating the effects of RS versus control on oxidative stress biomarkers. Outcomes included: MDA **(A)** and SOD **(B)**. MDA, malondialdehyde; SOD, superoxide dismutase.

Figure 7 presented the changes in MDA and SOD levels across all subjects. Based on six trials, RS consumption did not significantly reduce MDA levels (SMD = −0.30, 95% CI: −0.65 to 0.05, *p* = 0.09), with high heterogeneity (*I*^2^ = 65%). In contrast, data from five trials showed a significant improvement in SOD activity following RS intake (SMD = 0.29, 95% CI: 0.08–0.51, *p* = 0.008), with no observed heterogeneity (*I*^2^ = 0%).

### 3.9 Subgroup analysis

#### 3.9.1 Subgroup analysis on anthropometric parameters

The findings from the subgroup analysis of anthropometric parameters were detailed in Supplementary Table 5. In the case of BW, a significant reduction was observed in the subgroup with NAFLD, the subgroup where the mean participant age was below 45 years, the subgroup where the mean BMI was less than 30 kg/m^2^, and the cohort with a higher dosage of 30 g/day or more. A more significant decrease in BMI was demonstrated in the subgroup with the high score of the Jadad scale, the subgroup with prediabetes or T2DM and the subgroup with NAFLD. A significant reduction in WC was observed in the subgroup consuming 30 g/day or more and the subgroup with NAFLD.

Regarding body shape, the subgroup from outside western developed countries, with a mean age under 45 years old as well as a mean BMI below 30 kg/m^2^, and the subgroup with NAFLD observed a significant decrease in FM. There was a significant reduction in body fat percentage in the subgroup that achieved a high score on the Jadad scale, with a mean participant age of less than 45 years and a mean BMI of less than 30 kg/m^2^, the subgroup where RS was administered as a supplement, there was a reduction in body fat percentage, and the subgroup with NAFLD.

In terms of blood pressure, a significant reduction in SBP level was observed in the NAFLD subgroup, the subgroup using RS as a supplement, and the subgroup receiving at least 30 g/day of RS. DBP level was significantly decreased in the NAFLD subgroup, the subgroup using RS as a supplement, the subgroup taking a dose of 30 g/day or more, and those with intervention lasting 8 weeks or longer.

#### 3.9.2 Subgroup analysis on glycemic profiles

The findings from the subgroup analysis of glycemic profiles were summarized in Supplementary Table 6. For FINS, significant reductions were observed across different disease subgroups. Specifically, the intake of RS resulted in a statistically significant reduction in FINS levels among participants with overweight or obesity, those with prediabetes or T2DM, and those with NAFLD. RS significantly enhanced HOMA-IR in the subgroup characterized by a high score and the subgroup with NAFLD. A substantial reduction of HOMA-β was reported in overweight or obese participants.

#### 3.9.3 Subgroup analysis on lipid profiles

The findings from the subgroup analysis of lipid profiles were detailed in Supplementary Table 7. A notable decrease in serum TG levels was observed among participants with NAFLD. Regarding HDL-C, a modest yet statistically significant increase was observed in the subgroup that attained a high score on the Jadad scale as well as in the subgroup comprising participants with a mean age below than 45 years.

#### 3.9.4 Subgroup analysis on inflammatory factors

The findings from subgroup analysis of inflammatory markers were presented in Supplementary Table 8. RS consumption notably reduced TNF-α levels across various subgroups: low ROB, unclear ROB, overweight or obese participants and NAFLD. No significant differences were observed for IL-6 levels across any subgroups.

#### 3.9.5 Subgroup analysis on oxidative stress biomarkers

The subgroup analysis primarily focused on MDA in the evaluation of oxidative stress biomarkers, as detailed in Supplementary Table 9. RS consumption notably reduced TNF-α levels across various subgroups: low ROB, unclear ROB, high score of the Jadad scale, other regions outside western developed countries, hyperlipidemia, and a mean age 45 years or more as well as parallel assignment.

### 3.10 Sensitivity analysis and publication bias

Sensitivity analysis indicated that the results were robust in the effect sizes, including HC, TC, LDL-C and SOD. Significant differences yet high heterogeneity persisted in the effect sizes, including WC, FINS, HOMA-IR, and TNF-α. The effect sizes of HDL-C and MDA newly demonstrated a significant enhancement after excluding the studies judges with a high ROB (Supplementary Table 10).

Publication bias was assessed using Begg’s and Egger’s tests, with results summarized in Supplementary Table 11. Begg’s test indicated potential publication bias for HbA1c (P_Begg_ = 0.027). Egger’s test detected evidence of publication bias for WC (P_Egger_ = 0.007), DBP (P_Egger_ = 0.046), FBG (P_Egger_ = 0.033), and TG (P_Egger_ < 0.001). Corresponding funnel plots and bias assessments for these five outcomes were presented in Supplementary Figures 1–5. The Trim and Fill methods were applied to WC, DBP, FBG, and TG to adjust for potential bias. For WC, inclusion of three imputed studies yielded a non-significant change in the overall effect size (MD = −4.159 cm, 95% CI: −6.085 to −2.233, *p* < 0.001). For FBG, the adjusted meta-analysis remained robust, with no additional studies imputed and no change in significance (MD = 0.019 mmol/L, 95% CI: −0.055 to 0.094, *p* = 0.608). However, significant corrected effect sizes were observed after adjustment in DBP (MD = −2.981 mmHg, 95% CI: −4.825 to −1.136, *p* = 0.002; 3 imputed studies) and TG (MD = −0.466 mmol/L, 95% CI: −0.657 to −0.276, p < 0.001; 7 imputed studies).

## 4 Discussion

This present systematic review and meta-analysis was the first to assess the effects of RS consumption on anthropometric parameters and serum biomarkers in the adults with MetS-related risks. A total of 23 RCTs were subjected to quality assessment, while 20 trials were incorporated into for meta-analysis. Our findings revealed the effects of RS consumption on HC, TC, LDL-C and SOD with low heterogeneity. The low heterogeneity was attributed to several factors such as the high quality of study design, participants with younger age and overweight, a supplement as delivery, a dose of up to 30 g/day, and lasting over 8 weeks.

### 4.1 Effect sizes of anthropometric and serum parameters

The present review showed RS lowered WC and HC by 2.58 cm and 1.83 cm, respectively. Between the two, WC serves as an indicator of adipose distribution, and abdominal obesity, as determined by WC, is one component of MetS (46). The prior review consistently demonstrated the effect of nondigestible fermentable carbohydrates intake on WC (47). The decrease in WC indirectly indicated a reduction in central adiposity, a pattern of fat distribution, similarly observed in murine models administered with RS (48).

In the current review, neither SBP nor DBP was reduced by RS consumption. However, the findings indicated a reduced impact of RS on DBP following the application of the simulated method. To the best of our knowledge, there is a lack of research investigating the effect of RS on hypertension trials or systematic reviews related to blood pressure. A prior review regarding on the effect of fermentable carbohydrates intake on SBP in adults with overweight and obesity (47). The study on individuals with NAFLD revealed the a significant reduction in SBP and DBP following the consumption of RS for 3 months (11). Another study demonstrated a lessened result of DBP after consuming RS for 12 weeks (35). A possible explanation was that protein intake, compared to carbohydrate intake, was consistently associated with beneficial effect on blood pressure (49).

The meta-analysis showed that consumption of RS improved insulin sensitivity, as indicated by measures such as FINS, HOMA-IR, and HbA1c. Elevated blood glucose levels prompts the secretion of insulin by pancreatic islet beta cells. Insulin resistance is characterized by compensatory hyperinsulinemia. Previous systematic reviews have supported our findings regarding the reduction of FINS level through RS consumption in populations with overweight, obesity, MetS, prediabetes, and T2DM (8, 16, 50). HOMA-IR was used to assess the insulin resistance. Two reviews documented the same results of HOMA-IR (14, 16). The anticipated reduction in FBG levels due to RS intake was not observed. Previous studies demonstrated that RS reduced glucose levels during the postprandial period rather than during fasting (51–53). As for HbA1c, it is conventionally employed as a mean indicator of blood glucose levels to assess glycemic control. We found that HbA1c was lowered by 0.14% after RS consumption. The result was consistent with the preliminary review concerning overweight, obesity, and MetS (8, 16).

The current review identified a reduction in TC and LDL-C concentrations by 0.20 mmol/L and 0.11 mmol/L, respectively, after RS consumption. The effects of RS intake on two biomarkers were consistent across healthy individuals, as well as patients with overweight, obesity and MetS in the previous reviews (16, 47, 54). In a double-blind parallel RCT conducted by Ni et al. (11), it was reported that the intake of RS for 4 months improved TG and HDL-C concentrations. Another study published by Gargari et al. (41) showed that the intake of RS for 8 weeks increased HDL-C levels.

Three inflammatory indicators, including TNF-α, IL-6, and hs-CRP, were reported to be associated with component score of MetS, according to the previous review (55). In the current review, it was demonstrated that the intake of RS led to a reduction in TNF-α levels, while no significant effect was observed on IL-6 levels. These findings are consistent with those reported in previous systematic reviews (16). However, two reviews, which included both healthy subjects and those with diseases and the intervention of RS and resistant dextrin, reported improvements in two biomarkers (56, 57). Previous reviews noted the minimal impact of RS consumption on CRP levels (16–18, 56, 57). Some report indicated hs-CRP is more precise and suitable for detecting elevated cardiometabolic risks than the conventional measurement (58). In the present review, we investigated the impact of RS on hs-CRP levels; however, the finding indicated little effect of RS with low heterogeneity.

As for oxidative stress biomarkers, the concentration of SOD increased, while MDA levels remained unchanged following the consumption of RS. A prior review has provided evidence of improvements in the two biomarkers within the included trials, following interventions with RS and resistant dextrin, in the context of diseases involving chronic kidney disease (17), while another review demonstrated the negative effects of RS intake on the two biomarkers (57). SOD is an enzyme that protects by eliminating superoxide free radicals and their metabolic byproducts. The SOD gene has been reported to be associated with various metabolic disorders (59–61). MDA is produced as a result of lipid peroxidation during metabolic stress. Glucolipotoxicity linked to MetS results in higher levels of plasma MDA (62). Both two reviews reported an enhanced serum level of total antioxidant capacity (TAC), although they exhibited high heterogeneity, including overlapping trials (17, 57). TAC serves as a measure reflecting overall antioxidant buffering power in the body. Conversely, a well-established study indicated that plasma TAC was unable to predict components of cardiometabolic risk, possibly due to the activation of compensatory mechanisms under physiological conditions (63).

Four studies were excluded from the meta-analysis. Of these, three did not provide baseline information, and one concentrated on postprandial changes in serum glucose and insulin. Despite the lack of baseline data, the study by Bodinham et al. (29) demonstrated a reduction in FINS, while Bodinham et al. (30) reported a decrease in TNF-α. Additionally, Robertson et al. (28) observed reductions in FBG, FINS, and HOMA-IR. In contrast, Maziarz et al. (33) found no significant between-group differences in biomarkers.

### 4.2 Potential factors of heterogeneity

The classification of RS types was considered to introduce bias, as various *in vitro* and animal studies have demonstrated that different RS types exhibit variations in digestibility, fermentation rates, and SCFA profiles (21, 64, 65). Only RS2 and RS3 were included in the present meta-analysis, and most (16 of 20) of the trials used RS2. Two records from a well-established study investigating the effect of RS4-enriched flour on metabolic risks were identified, and a significant reduction of TC was observed (66, 67). However, these records were excluded from the current review due to the consumption being ad libitum without a specified dosage, and the inclusion of both healthy individuals and those with MetS. In the present review, subgroup analysis revealed there were no differences between RS2 and RS3 across any parameters. Pugh et al. (68) identified a statistically significant effect of RS2 on FINS level in comparison to RS1. However, the content of RS was not reported in RS1 subgroup, making it difficult determine the differences among RS type. Furthermore, Yuan et al. (54) indicated that RS2, rather than RS3, was associated with the regulation of serum TC and TG, regardless of participants’ health status. We considered there was substantial heterogeneity within the subgroups of two RS types, suggesting that other factors influenced the RS effects.

Food matrix has been considered as a critical contributor to heterogeneity, since starch processing changes not only properties, such as digestibility, gelatinization, retrogradation, crystallinity, amylose to amylopectin ratio, but also bioavailability in recent reports (69–71). Most (13 of 20) of trials encompassed in the current review administered RS in the form of a powdered supplement, thereby preserving the integrity of the heat-labile RS. Conversely, seven studies incorporated RS into food matrices that were subsequently subjected to cooking or baking processes, likely leading to a diminishment in RS content. Moreover, the incorporation of RS flour into food matrices, resulted in a reduced RS content, thereby leading to a decreased actual intake. Thus, the delivery method can affect RS intake and potentially alter outcomes. In the subgroup receiving the supplement delivery, a higher content ranging from 34 to 60% and a dose between 6 g and 40 g were achieved, compared to the subgroup utilizing food matrices, which exhibited a content range of 3.1–21.2% and a dose of 4–30.9 g. Most of RS-rich powders using in the included studies were commercially manufactured Hi-maize 260. In the subgroup receiving RS as a supplement, body fat percentage, SBP, and DBP significantly decreased, but these effects were absent when RS was part of food matrices. Wei et al. (57) reported the supplement form influenced the RS effects on inflammation biomarkers. Unfortunately, due to the limited information provided in the included studies, details regarding the properties of intervention, such as amylose to amylopectin ratio or crystallinity, were not available within the context of the articles.

Subgroup analysis on the dose and the duration of intervention was conducted. The result showed that consuming 30 g/day of RS improved anthropometric parameters, such as BW, WC, body fat percentage, SBP and DBP, significantly. Johnston et al. (15) reported that the RS supplement was integrated into the daily food twice per day. Li et al. (12) proposed that the RS supplement be administered as a powder dissolved in 300 mL of water. Meanwhile, Maziarz et al. (33) ensured an adequate RS content by incorporating RS powder into muffins. Two reviews set the dose cut-off at 20 g/day (18, 54), but only one reported its role of the mediator in RS effect on TC level. We considered the limited role of the dosage appeared in previous reviews, as it did not meet the recommended dietary fiber intake (72). As for the duration, according to published reviews, RS has been demonstrated to reduce serum inflammation biomarkers for a loner duration (56, 57). Here, we found the subgroup with over 8 weeks of intervention showed greater reductions in body fat percentage and DBP compared to those with less than 8 weeks.

We conducted subgroup analysis on participants characteristics, such as region, disease, mean age and mean BMI. The world’s division into western and eastern regions influenced the RS effects on glycemic, lipid and inflammatory profiles, as noted in two previous reviews (16, 57). We found that the RS consumption significantly decreased the levels of FM and MDA in the participants from outside western developed region. The RS effects in the current review were partly influenced by the disease profile. The result was cautiously presented, as only single trial with disease, such as hyperlipidemia, or NAFLD, was included in the review. In the younger subgroup, RS consumption significantly affected BW, FM, body fat percentage, and HDL-C, while it reduced MDA level in the middle and elder subgroup. Previous reviews also noted significant RS effects on glycemic, lipid, and inflammatory biomarkers in the younger subgroup (16, 57). RS consumption led to reductions in BW, FM and body fat percentage was decreased among overweight participants. We concerned RS consumption, as the supplement intervention, exhibited greater sensitivity in younger individuals and those with a lower BMI.

Subgroup analysis on study design based on Cochrane ROB tool, the score of the Jadad scale, and assignment, was explored as well. The levels of TNF-α and MDA were significantly improved by RS intake in the subgroups of trials judged as low or unclear ROB, while the parameters of BMI, body fat percentage, HOMA-IR, HDL-C, and MDA were enhanced in the subgroups of trials achieved a higher score of the Jadad scale. Both quality assessment tools were employed in systematic reviews. The first tool was evaluated across a broad range of aspects, whereas the second scale emphasized rigorous randomization and blinding methods. The results of the subgroup analysis indicated the necessity for a high-quality study design to research on the clinical functionalities of RS. Differences of assignment was only influenced the results of MDA. RS intervention is highly susceptible to carryover effects due to microbiota adaptation. Therefore, we recommend that a longer washout period be implemented in crossover trials.

### 4.3 Biological mechanisms

In relation to mechanisms of regulating anthropometric and serum biomarkers, the functional benefits of RS consumption can be attributed to either its physical–chemical structure or its biological impact.

Furthermore, the application of RS enhances the water absorption capacity of doughs and diminishes the viscoelastic properties of foods (73), thereby potentially augmenting satiety and decreasing the intake of digestible carbohydrates. RS-containing diets notably decreased energy intake and BW *in vivo* (74). In human, the preliminary review gave inconclusive evidence that RS had effects on appetite, hunger, food intake, satiety (75). The malabsorption of RS in the small intestine leads to a reduction in the glycemic index following ingestion, resulting in minimal fluctuations in postprandial blood glucose levels, which effectively enhances glucose tolerance (76). RS3 can be fermented in the colon and may bind bile salts, reducing bile acid reabsorption in the ileum, stimulating hepatic bile acid production, and increasing cholesterol use (77).

The mechanism of RS consumption may be partially attributed to the indirect effects mediated by the gut microbiome and the production of SCFAs. Indigestible RS are fermented by the predominant beneficial gut microbiota, such as Bifidobacterium, Lactobacilli, Ruminococcus, Roseburia, Eubacterium, Faecalibacterium, and Clostridium among others (78–80). Bifidobacterium, Faecalibacterium and Ruminococcus were typically increased after RS2, RS3, and RS4 supplementations, respectively, according to published trials (10, 37, 67). Fermentation of RS generates SCFAs including acetate, propionate, butyrate (81). RS can reduce appetite by enhancing the secretion of satiety-associated hormones like glucagon-like peptide-1 (GLP-1) and peptide YY (PYY) by SCFAs targeting G protein-coupled receptors (GPRs) (82). It was found that muffins with RS content enhanced satiety, prolonged digestion period, and benefited to weight loss (33). Replacing carbohydrates with RS led to a notable increase in postprandial fat oxidation (83). Additionally, the fermented SCFAs shifted metabolic pathway from lipogenesis to fat oxidation by activating peroxisome proliferator-activated receptor-γ (84). These findings suggest that RS may contribute to reducing fat accumulation.

Fermented SCFAs could reduce the gastric motility and intestinal glucose transport capacity by stimulating PYY and GLP-1, respectively (85, 86). The Dose-dependent Kudzu RS enhanced insulin sensitivity and reinstated the hepatic expression of insulin receptor substrate 1 and glucose transporter 4, both of which contributed to insulin efficacy and glucose homeostasis, in T2DM mice (87). Serum SCFAs have been shown to improve insulin sensitivity by interacting with G protein-coupled receptors (GPRs) [61]. Butyrate has been shown to mitigate adipocyte inflammation by inhibiting the NOD-like receptor thermal protein domain associated protein 3 pathway [71]. Bacterial toxins, particularly lipopolysaccharides (LPS), which are integral components of Gram-negative bacteria, are primary contributors to the cause of low-grade chronic systemic inflammation (88). Gut microbiota-derived LPS affect intestinal permeability and tight junction (TJ) proteins by activating toll-like receptor 4-dependent pathways in intestine (89). Compromised intestinal permeability led to LPS translocation into the blood. Dietary RS addition increased propionate and butyrate and ameliorated LPS-induced inflammation during LPS challenge in vivo (90). Both SCFAs improve the intestinal barrier function by upregulating TJ proteins in epithelial intestine cells (91, 92). LPS induces inflammatory cytokines secretion, like TNF-α, by mediating inflammatory host cells, such as macrophage (93). Chronic inflammation induces lipid peroxidation through inhibition of catalase and glutamine peroxidase activities (94). SCFAs play protective role in inflammatory cytokines and oxidative stress (94, 95). However, there have been no reports demonstrating that SCFAs fermented by RS modulate inflammation and oxidative stress. The probable mechanism is that the fermentation of RS reduces the diversity and expression of hazard gut microbiota (7).

Dietary RS mediates bile acid (BA) metabolism and promotes GLP-1 secretion by targeting Takeda GPR 5 (96). Lei et al. demonstrated lotus seed RS lowered serum lipid by promoting BAs excretion and regulating gut microbiome (97). Included trials reported distinct changes of BA metabolism after RS2 and RS3 (10–12).

### 4.4 Strengths and limitations

We implemented strict eligibility criteria for including RCTs, standardized algorithms and uniformity in the units before calculating effect sizes. The heterogeneity was evaluated from the variety of aspects, such as RS type, intervention settings, participant characteristics, and study design. We employed a variety of measurement tools for qualitive assessments, sensitivity analysis and publication bias.

However, our study still had several limitations. Additional detailed parameters of RS, such as crystallinity, amylose to amylopectin ratio, gelatinization, and retrogradation, were not collected, even though these factors potentially influenced the observed effects. It was hard to draw a solid conclusion due to the absence of proposed mechanisms in the majority of the included studies. The present review focused on the limited scope of adults excluding children or adolescents. We included the participants with metabolic risks, including overweight, obesity, MetS, prediabetes, T2DM, hyperlipidemia, and NAFLD. While these conditions exhibited similarities in terms of pathogenesis and symptoms, it was important to acknowledge the distinctions that existed between them.

## 5 Conclusion

In conclusion, this meta-analysis demonstrated the beneficial effects of RS on HC, TC, LDL-C, and SOD in the management of adults with embolic syndrome-related risks. The inconclusive evidence showed the effects of RS on WC, FINS, HOMA-IR, TNF-α, and HDL-C and MDA. A dose of at least 30 g/day and duration over 8 weeks partly help with a positive observation of RS. The participants with younger age and overweight, and the high-quality study design are recommended. Future studies are warranted with the assessments of the properties, delivery mode, gut microbial composition, and metabolome, for further insights.

## Data Availability

The original contributions presented in the study are included in the article/Supplementary material, further inquiries can be directed to the corresponding authors.

## Footnotes

^1^ https://www.statstodo.com/Contact.php
