# Supplementary material for: Effects of resistant starch consumption on anthropometric and serum parameters in adults with metabolic syndrome-related risks: a systematic review and meta-analysis
Source: Front Nutr. 2025 Sep 25;12:1655664. doi: 10.3389/fnut.2025.1655664 (PMC12507596; doi:10.3389/fnut.2025.1655664)
Supplement: Supplementary file 1 [file Data_Sheet_1.docx]

Supplementary Material

# Supplementary Figures and Tables

## Supplementary Figures


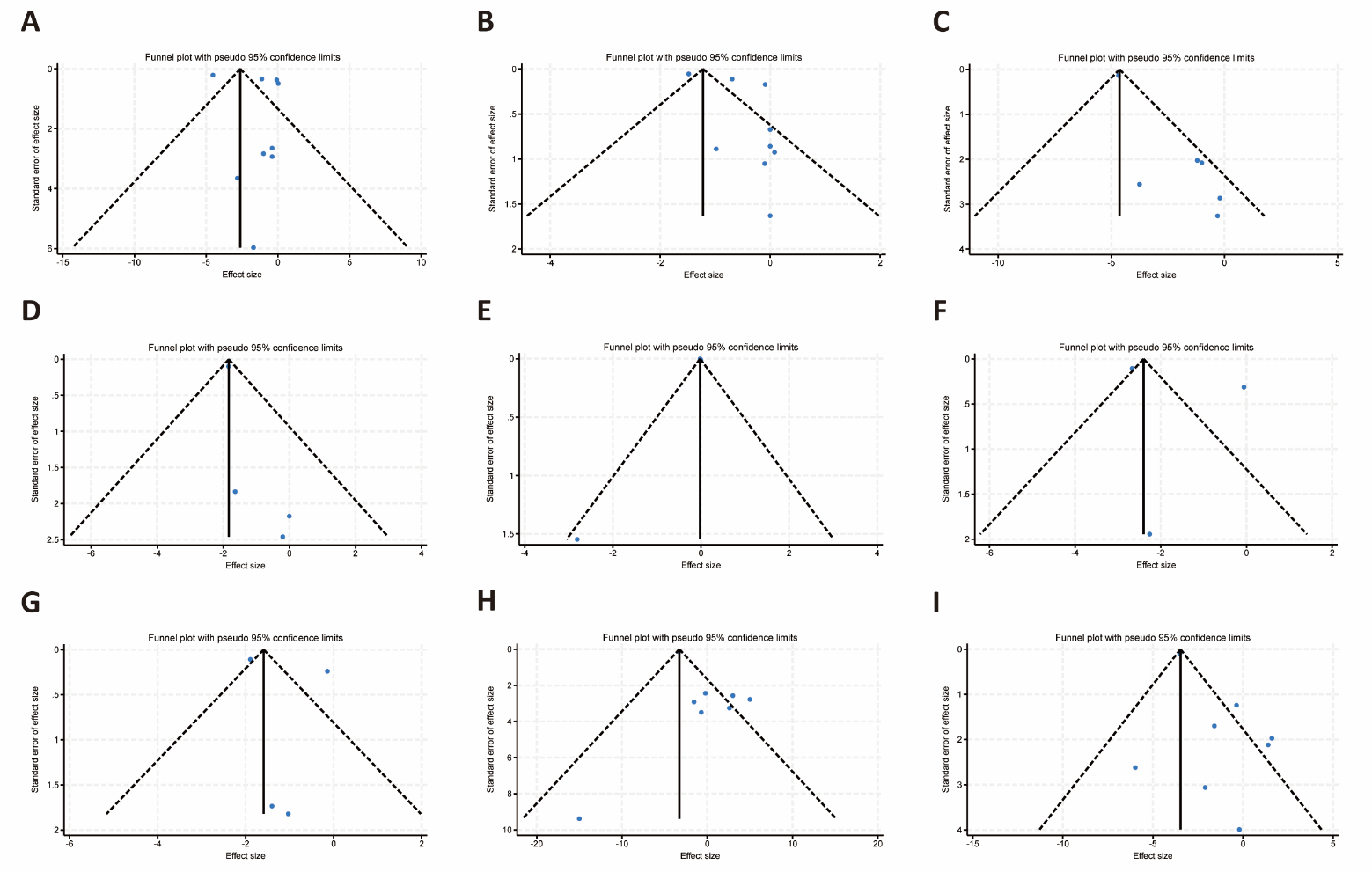


**Supplementary Figure 1.** Funnel plots of anthropometric parameters were generated for BW (A), BMI (B), WC (C), HC (D), waist-and-hip ratio (E), FM (F), body fat percentage (G), SBP (H), and DBP (I). Abbreviation: BW, body weight; BMI, body mass index; WC, waist circumference; HC, hip circumference; WHR, waist-to-hip ratio; FM, fat mass; DBP, diastolic blood pressure; SBP, systolic blood pressure.


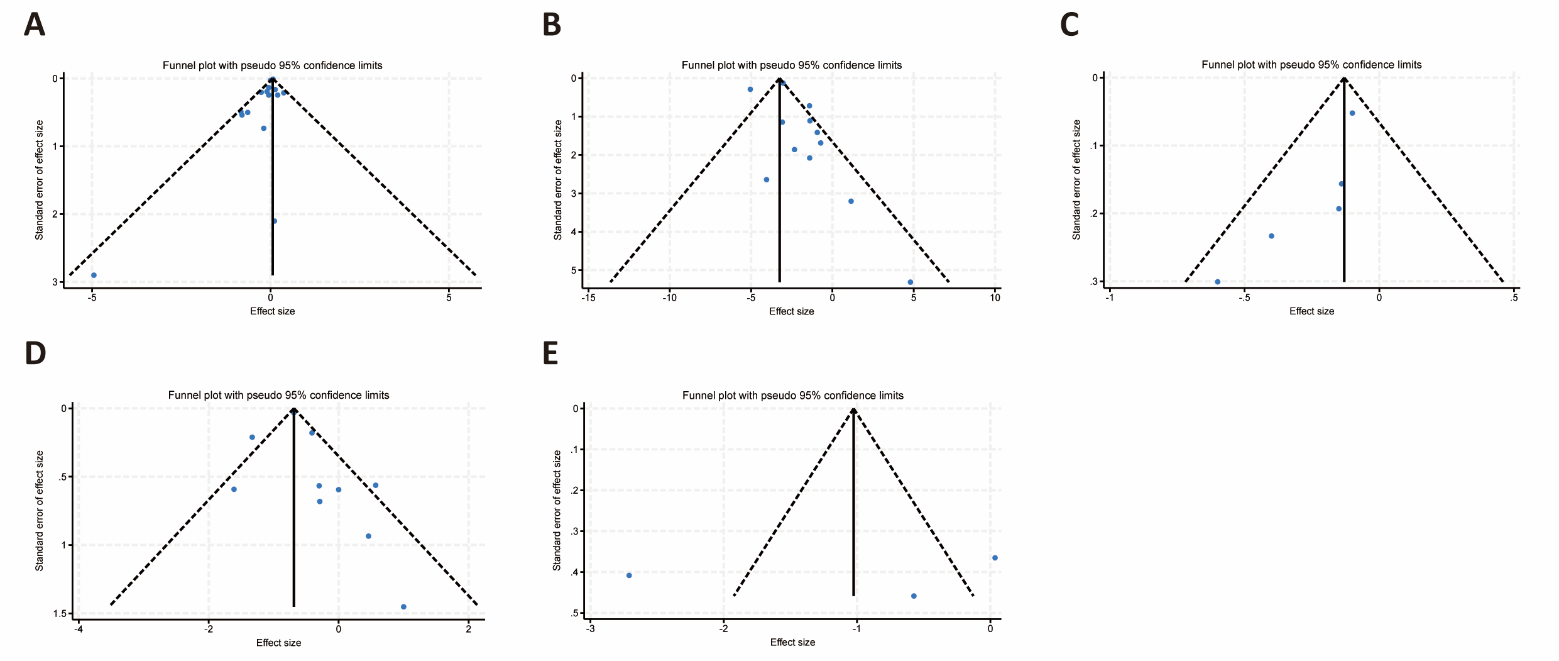


**Supplementary Figure 2.** Funnel plots of glycemic profiles were generated for FBG (A), FINS (B), HbA1c (C), HOMA-IR (D), and HOMA-β (E). Abbreviation: FBG, fasting blood glucose; FINS, fasting insulin; HbA1c, glycated hemoglobin; HOMA-IR, homeostatic model assessment of insulin resistance; HOMA-β, homeostatic model assessment of beta-cell function.


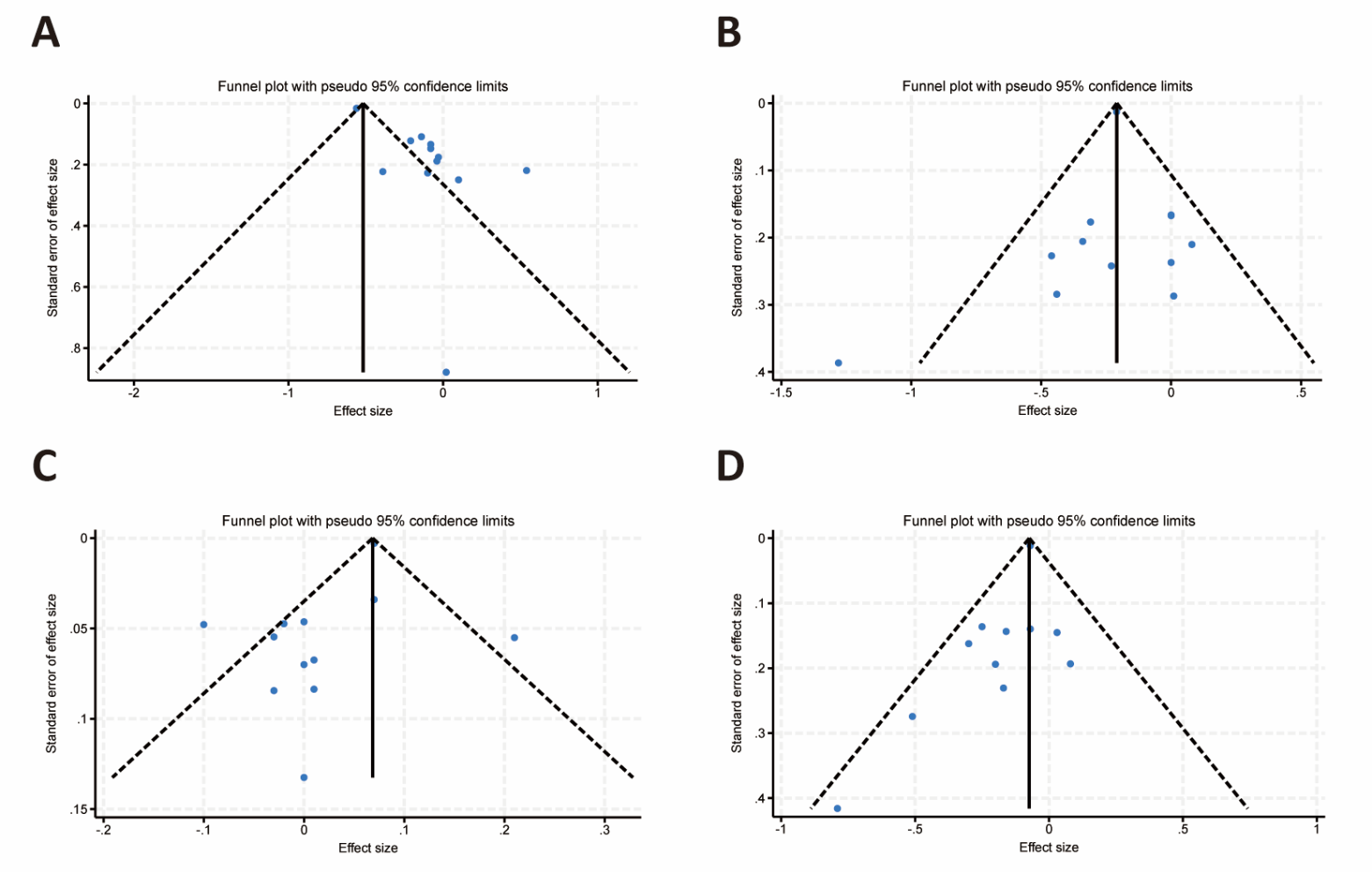


**Supplementary Figure 3.** Funnel plots of lipid profiles were generated for TG (A), TC (B), HDL-C (C), and LDL-C (D). Abbreviation: TG, triglycerides; TC, total cholesterol; HDL-C, high-density lipoprotein cholesterol; LDL-C, low-density lipoprotein cholesterol.


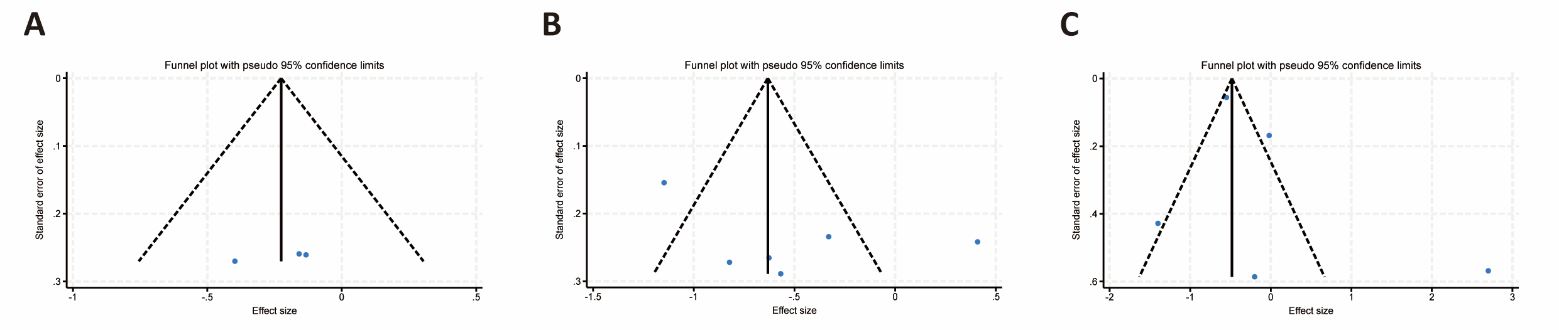


**Supplementary Figure 4.** Funnel plots of inflammatory factors were generated for hs-CRP (A), TNF-α (B), and IL-6 (C). Abbreviation: hs-CRP, high-sensitivity C-reactive protein; TNF-α, tumor necrosis factor-alpha; IL-6, interleukin-6.


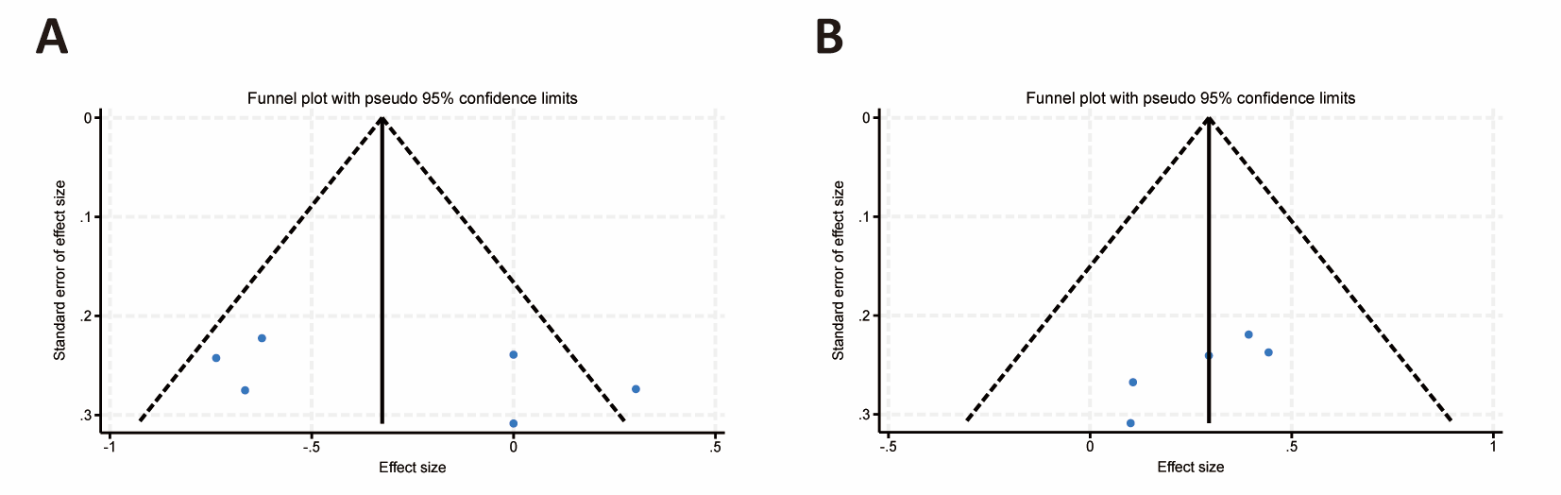


**Supplementary Figure 5.** Funnel plots of oxidative stress biomarkers were generated for MDA (A) and SOD (B). Abbreviation: MDA, malondialdehyde; SOD, superoxide dismutase.

## Supplementary Tables

**Supplementary Table 1.** The Explicit Search Strategy Used Per Database

| Databases | Search strategy |
| --- | --- |
| PubMed | ​(("Resistant Starch"[Mesh]) OR ("resistant starch*"[Title]) OR ("resistant starch*"[Title/Abstract])) AND (("effect*"[Title/Abstract]) OR ("efficac*"[Title/Abstract]) OR ("function*"[Title/Abstract]) OR ("benefi*"[Title/Abstract]) OR ("prebiotic*"[Title/Abstract]) OR ("impact*"[Title/Abstract]) OR ("induc*"[Title/Abstract])) |
| Web of Science | ​(TI = ("resistant starch*")) AND (TS = (("effect*") OR ("efficac*") OR ("function*") OR ("benefi*") OR ("prebiotic*") OR ("impact*") OR ("induc*"))) |
| Chocrane Library | #1 MeSH descriptor: [Resistant Starch] explode all trees #2 (resistant starch*):ti #3 #1 OR #2 #4 (impact*):ti,ab,kw OR (induc*):ti,ab,kw #5 (effect*):ti,ab,kw OR (efficac*):ti,ab,kw OR (function*):ti,ab,kw OR (benefi*):ti,ab,kw OR (prebiotic*):ti,ab,kw #6 #4 OR #5 #7 #3 AND #6 |
| Embase | #1 resistant starch'/exp #2 'resistant starch*':ti #3 #1 OR #2 #4 effect*:ab. i OR eficac*:ab,ti OR function*:ab,ti OR benef*:ab,ti OR prebiotic*:ab,ti OR impact*:ab,ti OR induc*:ab,.ti #5 #3 AND #4 |
| Scopus | TITLE ( resistant AND starch* ) AND ( TITLE-ABS-KEY ( effect* ) OR TITLE-ABS-KEY ( efficac* ) OR TITLE-ABS-KEY ( function* ) OR TITLE-ABS-KEY ( benefi* ) OR TITLE-ABS-KEY ( prebiotic* ) OR TITLE-ABS-KEY ( impact* ) OR TITLE-ABS-KEY ( induc* ) ) |

**Supplementary Table 2.** The properties of Resistant Starch in Included Studies

| Study | Intervention substance (dose, g/day) | RS type | Source | Purity (%) | Content analytical method | Delivery mode | Feasibility |
| --- | --- | --- | --- | --- | --- | --- | --- |
| Ble-Castillo 2010 | Native banana starch (8.16/24) | RS2 | Banana | 34 | Goni method | Supplement | Starch dissolved in 240 mL of water per day |
| Bodinham 2012 | Hi-maize 260 (40/67) | RS2 | Maize | 60 | AOAC 991.43 | Supplement | Starch supplied to ready-to-use sachets that simply required mixing into a cold liquid |
| Bodinham 2014 | Hi-maize 260 (40/67) | RS2 | Maize | 60 | AOAC 991.43 | Supplement | Starch supplied to ready-to-use sachets that were mixed into a beverage |
| Cao 2022 | Retrograded potato (17.5/350) | RS3 | Potato | 5 | AOAC 2002.02 | Food | Dietary Guidelines for Americans with potato |
| Costa 2019 | Native banana biomass (4.5/40) | RS2 | Banana | 11.3 | Goni method | Food | Added to any food preparation without heating |
| Dainty 2016 | Bagel containing Hi-maize 260 (25.4/119.8) | RS2 | Maize | 21.2 | AOAC 991.43 | Food | Consumption within bagel |
| Eshghi 2019 | Hi-maize 260 (8.1/13.5) | RS2 | Maize | 60 | AOAC 991.43 | Supplement | Starch supplement |
| Gargari 2015 | Hi-maize 260 (6/10) | RS2 | Maize | 60 | AOAC 991.43 | Supplement | Supplements divided into two packages of 5 g for lunch and dinner suspended in a cup of water |
| Johnston 2010 | Hi-maize 260 (40/67) | RS2 | Maize | 60 | AOAC 991.43 | Supplement | Instructed to take two sachets of starch per day incorporated into their daily foods |
| Karimi 2016 | Hi-maize 260 (6/10) | RS2 | Maize | 60 | AOAC 991.43 | Supplement | Powdered |
| Kwak 2012 | Rice containing maize RS (6.5/210) | RS2 | Maize | 3.1 | Unknown | Food | Containing in rice for any of three main meals |
| Li 2024 | Hi-maize 260 (40/91.2) | RS2 | Maize | 43.9 | AOAC 991.43 | Supplement | Provided as powder in pre-packaged sachets to be mixed with 300 ml water |
| Maki 2012 (1) | Hi-maize 260 (18/30) | RS2 | Maize | 60 | AOAC 991.43 | Supplement | Provided as starch in individually packaged, ready-to-use sachets that could be mixed into cold or room-temperature beverages or foods |
| Maki 2012 (2) | Hi-maize 260 (9/15) | RS2 | Maize | 60 | AOAC 991.43 | Supplement | Provided as starch in individually packaged, ready-to-use sachets that could be mixed into cold or room-temperature beverages or foods |

| Study | Intervention substance (dose, g/day) | RS type | Source | Purity (%) | Content analytical method | Delivery mode | Feasibility |
| --- | --- | --- | --- | --- | --- | --- | --- |
| Maziarz 2017 | Muffins containing Hi-maize 260 (30.9/180) | RS2 | Maize | 16.7 | AOAC 991.43 | Food | Muffins |
| Meng 2019 | High RS, low-protein flour (17.41/50) | RS2 | Unknown | 34.8 | Unknown | Supplement | Replaced a common staple of equal quality at lunch and dinner |
| Miao 2024 | Canna edulis resistant starch (9.6/20) | RS3 | Canna edulis | 40 | AOAC 991.43 | Supplement | Take two packets of 10g RS powder each day |
| Ni 2023 | Hi-maize 260 (40/91.2) | RS2 | Maize | 43.9 | AOAC 991.43 | Supplement | Starch consumption |
| Park 2004 | Retrograded maize RS (24/40) | RS3 | Maize | 60 | Total dietary fiber determination kit | Supplement | Starch supplements in 250mL of water each day between 3-5 p.m. |
| Penn-Marshall 2010 | Bread containing Hi-maize 260 (12.4/121.8) | RS2 | Maize | 10.2 | AOAC 991.43 | Food | Three loaves of RS-containing baking bread |
| Peterson 2018 | Hi-maize 260 (27/45) | RS2 | Maize | 48 | AOAC 991.43 | Supplement | Added to the yogurt prior to consumption |
| Robertson 2012 | Hi-maize 260 (40/67) | RS2 | Maize | 60 | AOAC 991.43 | Supplement | Supplied as powder in ready-to-use sachets to mix in with food/drink |
| Schioldan 2018 | Healthy carbohydrate diet containing resistant starch (21/534) | RS2 | Maize and potato | 12 | AOAC 2002.02 | Food | Key foods containing heat-resistant high-amylose maize starch and raw potato-starch |

**Supplementary Table 2.** Cont.

Abbreviation: RS, resistant starch.

**Supplementary Table 3.** Quality Assessment of Included Studies Using the Cochrane Risk of Bias Assessment Tool

| Study | Random sequence generation | Allocation concealment | Blinding of participants and personnel | Blinding of outcome assessment | Incomplete outcome data | Selective reporting | Other bias | Overall judgement |
| --- | --- | --- | --- | --- | --- | --- | --- | --- |
| Ble-Castillo 2010 | Random | Unclear | Subject | Unclear | low | no | no | Unclear |
| Bodinham 2012 | Random | Unclear | Subject | Unclear | low | no | no | Unclear |
| Bodinham 2014 | Random | Unclear | Subject | Unclear | low | no | no | Unclear |
| Cao 2022 | Random | Unclear | Non-blinded | Blinded | low | no | no | High |
| Costa 2019 | Computer-generated random number | Unclear | Non-blinded | Blinded | low | no | no | High |
| Dainty 2016 | Random | Unclear | Double-blinded | Unclear | low | no | no | Unclear |
| Eshghi 2019 | Computer-based randomization | Unclear | Double-blinded | Blinded | 22.20% | no | no | High |
| Gargari 2015 | Block randomization | Random Allocation Software | Triple-blinded | Blinded | low | no | no | Low |
| Johnston 2010 | Random | Unclear | Subject | Unclear | low | no | no | Unclear |
| Karimi 2016 | Block randomization | Random Allocation Software | Triple-blinded | Blinded | low | no | no | Low |
| Kwak 2012 | random | Unclear | Double-blinded | Blinded | low | no | no | Unclear |
| Li 2024 | Computer-based randomization | Sealed bag | Double-blinded | Blinded | low | no | no | Low |
| Maki 2012 (1) | Random | Unclear | Double-blinded | Blinded | low | no | no | Unclear |
| Maki 2012 (2) | Random | Unclear | Double-blinded | Blinded | low | no | no | Unclear |
| Maziarz 2017 | Computer-generated random number | Unclear | Double-blinded | Unclear | 28% | no | no | High |
| Meng 2019 | Computer-generated random sequences | not concealed | Non-blinded | Unclear | low | no | no | High |
| Miao 2024 | Computer-generated random schedule | Concealed | Double-blinded | Unclear | low | no | no | Unclear |
| Ni 2023 | Random | Sealed bag | Double-blinded | Unclear | low | no | no | Unclear |
| Park 2004 | Random | Unclear | Double-blinded | Unclear | low | no | no | Unclear |
| Penn-Marshall 2009 | Random | Unclear | Double-blinded | Unclear | low | no | no | Unclear |
| Peterson 2018 | Random | Unclear | Double-blinded | Unclear | low | no | no | Unclear |
| Robertson 2012 | Random | Unclear | Subject | Unclear | low | no | no | Unclear |
| Schioldan 2018 | Random | Unclear | Double-blinded | Unclear | low | no | no | Unclear |
| Sunarti 2022 | Random | Unclear | Subject | Unclear | low | no | no | Unclear |

**Supplementary Table 4.** Quality Assessment of Included Studies Using the Jadad Scale

| Study | Radomization | Blinding | Dropouts | Total score |
| --- | --- | --- | --- | --- |
| Ble-Castillo 2010 | 1 | 0 | 1 | 2 |
| Bodinham 2012 | 1 | 0 | 1 | 2 |
| Bodinham 2014 | 1 | 0 | 1 | 2 |
| Cao 2022 | 1 | 0 | 1 | 2 |
| Costa 2019 | 2 | 0 | 1 | 3 |
| Dainty 2016 | 1 | 2 | 1 | 4 |
| Eshghi 2019 | 2 | 2 | 1 | 5 |
| Gargari 2015 | 1 | 2 | 1 | 4 |
| Johnston 2010 | 1 | 0 | 1 | 2 |
| Karimi 2016 | 1 | 2 | 1 | 4 |
| Kwak 2012 | 1 | 2 | 1 | 4 |
| Li 2024 | 2 | 2 | 1 | 5 |
| Maki 2012 (1) | 1 | 2 | 1 | 4 |
| Maki 2012 (2) | 1 | 2 | 1 | 4 |
| Maziarz 2017 | 2 | 2 | 1 | 5 |
| Meng 2019 | 2 | 0 | 1 | 3 |
| Miao 2024 | 2 | 2 | 1 | 5 |
| Ni 2023 | 1 | 2 | 1 | 4 |
| Park 2004 | 1 | 2 | 1 | 4 |
| Penn-Marshall 2009 | 1 | 2 | 1 | 4 |
| Peterson 2018 | 1 | 2 | 1 | 4 |
| Robertson 2012 | 1 | 0 | 1 | 2 |
| Schioldan 2018 | 1 | 2 | 1 | 4 |
| Sunarti 2022 | 1 | 0 | 1 | 2 |

**Supplementary Table 5.** Subgroup Analysis of Anthropometric Parameters

| Effect size | Subgroups | Trial count | Intervention group size | Control group size | MD (95% CI) | P for overall effect | I^2^ in subgroup (%) | Chi-square | P for subgroup differences |
| --- | --- | --- | --- | --- | --- | --- | --- | --- | --- |
| BW (kg) | RS type |  |  |  |  |  |  |  |  |
|  | RS2 | 8 | 320 | 308 | -1.41 (-3.54, 0.72) | 0.19 | 96 |  |  |
|  | RS3 | 1 | 12 | 13 | -0.40 (-5.59, 4.79) | 0.88 | - | 0.13 | 0.72 |
|  | Delivery mode |  |  |  |  |  |  |  |  |
|  | Supplement | 6 | 226 | 226 | -1.65 (-4.05, 0.76) | 0.18 | 96 |  |  |
|  | Food | 3 | 106 | 95 | -0.10 (-0.82, 0.61) | 0.78 | 0 | 1.46 | 0.23 |
|  | Dose |  |  |  |  |  |  |  |  |
|  | <30 g/day | 7 | 196 | 187 | -0.51 (-0.94, -0.08) | 0.02 | 0 |  |  |
|  | ≥30 g/day | 2 | 136 | 134 | -4.52 (-4.93, -4.11) | <0.00001 | 0 | 174.64 | <0.00001 |
|  | Duration |  |  |  |  |  |  |  |  |
|  | <8 weeks | 4 | 84 | 85 | -0.62 (-1.35, 0.12) | 0.10 | 31 |  |  |
|  | ≥8 weeks | 5 | 248 | 236 | -1.86 (-5.10, 1.39) | 0.26 | 95 | 0.53 | 0.47 |
|  | Region |  |  |  |  |  |  |  |  |
|  | Western developed | 3 | 48 | 49 | 0.02 (-0.94, 0.98) | 0.19 | 0 |  |  |
|  | Others | 1 | 284 | 272 | -1.60 (-3.89, 0.68) | 0.17 | 96 | 1.64 | 0.20 |
|  | Disease |  |  |  |  |  |  |  |  |
|  | Overweight or obesity | 4 | 95 | 96 | -0.12 (-0.82, 0.59) | 0.74 | 0 |  |  |
|  | MetS | 1 | 19 | 19 | -1.70 (-13.40, 10.00) | 0.78 | - |  |  |
|  | Prediabetes/T2DM | 3 | 119 | 109 | -0.63 (-1.56, -0.30) | 0.19 | 46 |  |  |
|  | NAFLD | 1 | 99 | 97 | -4.53 (-4.94, -4.12) | <0.00001 | - | 141.72 | <0.00001 |
|  | Mean age |  |  |  |  |  |  |  |  |
|  | <45 years | 4 | 169 | 168 | -3.16 (-5.54, -0.77) | 0.01 | 34 |  |  |
|  | ≥45 years | 5 | 163 | 153 | -0.46 (-1.07, 0.15) | 0.14 | 32 | 4.58 | 0.03 |
|  | Mean BMI |  |  |  |  |  |  |  |  |
|  | <30 kg/m^2^ | 3 | 148 | 147 | -3.82 (-5.95, -1.69) | 0.0004 | 24 |  |  |
|  | ≥30 kg/m^2^ | 5 | 165 | 155 | -0.46 (-1.06, 0.15) | 0.14 | 31 | 8.87 | 0.003 |
|  | ROB judgement |  |  |  |  |  |  |  |  |
|  | Low | 1 | 37 | 37 | -2.82 (-9.97, 4.33) | 0.44 | - |  |  |
|  | Unclear | 6 | 212 | 212 | -1.34 (-3.65, 0.97) | 0.26 | 97 |  |  |
|  | High | 2 | 83 | 77 | -1.33 (-3.37, 0.71) | 0.73 | 0 | 180.73 | 0.20 |

**Supplementary Table 5.** Cont.

| Effect size | Subgroups | Trial count | Intervention group size | Control group size | MD (95% CI) | P for overall effect | I^2^ in subgroup (%) | Chi-square | P for subgroup differences |
| --- | --- | --- | --- | --- | --- | --- | --- | --- | --- |
| BW (kg) | Jadad scale |  |  |  |  |  |  |  |  |
|  | Low score | 3 | 115 | 104 | -0.62 (-1.50, 0.25) | 0.16 | 54 |  |  |
|  | High score | 6 | 217 | 217 | -1.75 (-4.87, 1.37) | 0.27 | 93 | 0.46 | 0.50 |
|  | Assignment |  |  |  |  |  |  |  |  |
|  | Crossover | 5 | 117 | 118 | -1.12 (-1.77, -0.46) | 0.0008 | 0 |  |  |
|  | Parallel | 4 | 215 | 203 | -1.46 (-4.58, 1.65) | 0.36 | 98 | 0.05 | 0.83 |
| BMI (kg/m^2^) | RS type |  |  |  |  |  |  |  |  |
|  | RS2 | 7 | 282 | 269 | -0.58 (-1.22, 0.05) | 0.07 | 94 |  |  |
|  | RS3 | 2 | 39 | 40 | 0.00 (-1.49, 1.49) | 1.00 | 0 | 0.50 | 0.48 |
|  | Delivery mode |  |  |  |  |  |  |  |  |
|  | Supplement | 6 | 207 | 206 | -0.77 (-1.38, -0.15) | 0.01 | 90 |  |  |
|  | Food | 3 | 114 | 103 | -0.08 (-0.41, 0.25) | 0.62 | 0 | 3.72 | 0.05 |
|  | Dose |  |  |  |  |  |  |  |  |
|  | <30 g/day | 6 | 175 | 165 | -0.35 (-0.76, 0.06) | 0.15 | 47 |  |  |
|  | ≥30 g/day | 3 | 146 | 144 | -0.98 (-1.95, -0.02) | 0.05 | 61 | 1.40 | 0.24 |
|  | Duration |  |  |  |  |  |  |  |  |
|  | <8 weeks | 4 | 92 | 93 | -0.37 (-0.86, 0.12) | 0.14 | 67 |  |  |
|  | ≥8 weeks | 5 | 229 | 216 | -0.71 (-1.58, 0.15) | 0.11 | 58 | 0.46 | 0.50 |
|  | Region |  |  |  |  |  |  |  |  |
|  | Western developed | 2 | 37 | 37 | 0.00 (-1.22, 1.22) | 1.00 | 0 |  |  |
|  | Others | 7 | 284 | 272 | -0.60 (-1.24, 0.04) | 0.07 | 94 | 0.73 | 0.39 |
|  | Disease |  |  |  |  |  |  |  |  |
|  | Overweight/obesity | 4 | 95 | 94 | -0.12 (-0.44, 0.20) | 0.47 | 0 |  |  |
|  | Insulin resistance | 1 | 10 | 10 | 0.00 (-3.20, 3.20) | 1.00 | - |  |  |
|  | MetS | 1 | 27 | 27 | 0.00 (-0.22, 0.22) | 1.00 | - |  |  |
|  | Prediabetes/T2DM | 2 | 90 | 79 | -0.68 (-0.90, -0.46) | <0.00001 | 0 |  |  |
|  | NAFLD | 1 | 99 | 97 | -1.48 (-1.59, -1.37) | <0.00001 | - | 95.61 | <0.00001 |
|  | Mean age |  |  |  |  |  |  |  |  |
|  | <45 years | 5 | 196 | 195 | -1.00 (-1.72, -0.28) | 0.007 | 31 |  |  |

**Supplementary Table 5.** Cont.

| Effect size | Subgroups | Trial count | Intervention group size | Control group size | MD (95% CI) | P for overall effect | I^2^ in subgroup (%) | Chi-square | P for subgroup differences |
| --- | --- | --- | --- | --- | --- | --- | --- | --- | --- |
| BMI (kg/m^2^) | ≥45 years | 4 | 125 | 114 | -0.34 (-0.82, 0.14) | 0.17 | 68 | 2.26 | 0.13 |
|  | Mean BMI |  |  |  |  |  |  |  |  |
|  | <30 kg/m^2^ | 3 | 148 | 147 | -1.15 (-1.95, -0.35) | 0.005 | 39 |  |  |
|  | ≥30 kg/m^2^ | 6 | 173 | 162 | -0.34 (-0.75, 0.07) | 0.11 | 49 | 3.15 | 0.08 |
|  | ROB judgement |  |  |  |  |  |  |  |  |
|  | Low | 1 | 37 | 37 | -0.98 (-2.72, 0.76) | 0.27 | - |  |  |
|  | Unclear | 5 | 174 | 173 | -0.58 (-1.28, 0.11) | 0.10 | 96 |  |  |
|  | High | 3 | 110 | 99 | 0.00 (-1.25, 1.25) | 1.00 | 0 | 0.96 | 0.62 |
|  | Jadad scale |  |  |  |  |  |  |  |  |
|  | Low | 5 | 152 | 141 | -0.49 (-0.67, -0.31) | <0.00001 | 58 |  |  |
|  | High | 4 | 169 | 168 | -1.47 (-1.57, -1.36) | <0.00001 | 40 | 82.74 | <0.00001 |
|  | Assignment |  |  |  |  |  |  |  |  |
|  | Crossover | 5 | 125 | 126 | -0.67 (-0.89, -0.46) | <0.00001 | 0 |  |  |
|  | Parallel | 4 | 196 | 183 | -0.48 (-1.54, 0.58) | 0.37 | 95 | 0.12 | 0.73 |
| WC (cm) | RS type |  |  |  |  |  |  |  |  |
|  | RS2 | 4 | 219 | 206 | -3.22 (-5.42, -1.03) | 0.004 | 51 |  |  |
|  | RS3 | 2 | 39 | 40 | -0.24 (-4.46, 3.97) | 0.91 | 0 | 1.51 | 0.22 |
|  | Delivery mode |  |  |  |  |  |  |  |  |
|  | Supplement | 4 | 169 | 168 | -3.31 (-5.54, -1.07) | 0.004 | 45 |  |  |
|  | Food | 2 | 89 | 78 | -0.80 (-4.23, 2.64) | 0.65 | 0 | 1.44 | 0.23 |
|  | Dose |  |  |  |  |  |  |  |  |
|  | <30 g/day | 4 | 122 | 112 | -0.83 (-3.19, 1.52) | 0.49 | 0 |  |  |
|  | ≥30 g/day | 2 | 136 | 134 | -4.68 (-4.94, -4.41) | <0.00001 | 0 | 10.08 | 0.002 |
|  | Duration |  |  |  |  |  |  |  |  |
|  | <8 weeks | 2 | 39 | 40 | -0.24 (-4.46, 3.97) | 0.91 | 0 |  |  |
|  | ≥8 weeks | 4 | 219 | 206 | -3.22 (-5.42, -1.03) | 0.004 | 51 | 1.51 | 0.22 |
|  | Region |  |  |  |  |  |  |  |  |
|  | Western developed | 1 | 27 | 27 | -0.30 (-6.69, 6.09) | 0.93 | - |  |  |
|  | Others | 5 | 231 | 219 | -2.81 (-5.02, -0.61) | 0.01 | 53 | 0.53 | 0.47 |

**Supplementary Table 5.** Cont.

| Effect size | Subgroups | Trial count | Intervention group size | Control group size | MD (95% CI) | P for overall effect | I^2^ in subgroup (%) | Chi-square | P for subgroup differences |
| --- | --- | --- | --- | --- | --- | --- | --- | --- | --- |
| WC (cm) | Disease |  |  |  |  |  |  |  |  |
|  | Overweight/obesity | 3 | 70 | 71 | -1.72 (-4.44, 1.00) | 0.22 | 0 |  |  |
|  | MetS | 1 | 27 | 27 | -0.30 (-6.69, 6.09) | 0.93 | - |  |  |
|  | T2DM | 1 | 62 | 51 | -1.00 (-5.07, 3.07) | 0.63 | - |  |  |
|  | NAFLD | 1 | 99 | 97 | -4.68 (-4.95, -4.41) | <0.00001 | - | 9.34 | 0.03 |
|  | Mean age |  |  |  |  |  |  |  |  |
|  | <45 years | 5 | 196 | 195 | -2.97 (-5.17, -0.77) | 0.008 | 45 |  |  |
|  | ≥45 years | 1 | 62 | 51 | -1.00 (-5.07, 3.07) | 0.63 | - | 0.70 | 0.40 |
|  | Mean BMI |  |  |  |  |  |  |  |  |
|  | <30 kg/m^2^ | 3 | 148 | 147 | -4.17 (-5.97, -2.37) | <0.00001 | 22 |  |  |
|  | ≥30 kg/m^2^ | 3 | 110 | 99 | -0.97 (-3.57, 1.63) | 0.46 | 0 | 3.93 | 0.05 |
|  | ROB judgement |  |  |  |  |  |  |  |  |
|  | Low | 1 | 37 | 37 | -3.75 (-8.76, 1.26) | 0.14 | - |  |  |
|  | Unclear | 2 | 111 | 110 | -3.35 (-7.36, 0.65) | 0.10 | 59 |  |  |
|  | High | 3 | 110 | 99 | -0.97 (-3.57, 1.63) | 0.46 | 0 | 1.51 | 0.47 |
|  | Jadad scale |  |  |  |  |  |  |  |  |
|  | Low | 2 | 89 | 78 | -0.80 (-4.23, 2.64) | 0.65 | 0 |  |  |
|  | High | 4 | 169 | 168 | -3.31 (-5.54, -1.07) | 0.004 | 45 | 1.44 | 0.23 |
|  | Assignment |  |  |  |  |  |  |  |  |
|  | Crossover | 4 | 97 | 98 | -1.50 (-4.00, 1.00) | 0.24 | 0 |  |  |
|  | Parallel | 2 | 161 | 148 | -3.42 (-6.84, -0.01) | 0.05 | 68 | 0.49 | 0.37 |
| FM (kg) | RS type |  |  |  |  |  |  |  |  |
|  | RS2 | 3 | 165 | 164 | -1.55 (-3.80, 0.71) | 0.18 | 97 | - | - |
|  | Delivery mode |  |  |  |  |  |  |  |  |
|  | Supplement | 3 | 165 | 164 | -1.55 (-3.80, 0.71) | 0.18 | 97 | - | - |
|  | Dose |  |  |  |  |  |  |  |  |
|  | ≥30 g/day | 3 | 165 | 164 | -1.55 (-3.80, 0.71) | 0.18 | 97 | - | - |
|  | Duration |  |  |  |  |  |  |  |  |
|  | ≥8 weeks | 3 | 165 | 164 | -1.55 (-3.80, 0.71) | 0.18 | 97 | - | - |

**Supplementary Table 5.** Cont.

| Effect size | Subgroups | Trial count | Intervention group size | Control group size | MD (95% CI) | P for overall effect | I^2^ in subgroup (%) | Chi-square | P for subgroup differences |
| --- | --- | --- | --- | --- | --- | --- | --- | --- | --- |
| FM (kg) | Region |  |  |  |  |  |  |  |  |
|  | Western developed | 1 | 29 | 30 | -0.06 (-0.67, 0.55) | 0.85 | - |  |  |
|  | Others | 2 | 136 | 134 | -2.67 (-2.88, -2.46) | <0.00001 | 0 | 62.02 | <0.00001 |
|  | Disease |  |  |  |  |  |  |  |  |
|  | Overweight/obesity | 1 | 37 | 37 | -2.26 (-6.07, 1.55) | 0.25 | - |  |  |
|  | Prediabetes | 1 | 29 | 30 | -0.06 (-0.67, 0.55) | 0.85 | - |  |  |
|  | NAFLD | 1 | 99 | 97 | -2.67 (-2.88, -2.46) | <0.00001 | - | 62.06 | <0.00001 |
|  | Mean age |  |  |  |  |  |  |  |  |
|  | <45 years | 2 | 136 | 134 | -2.67 (-2.88, -2.46) | <0.00001 | 0 |  |  |
|  | ≥45 years | 1 | 29 | 30 | -0.06 (-0.67, 0.55) | 0.85 | - | 62.02 | <0.00001 |
|  | Mean BMI |  |  |  |  |  |  |  |  |
|  | <30 kg/m^2^ | 2 | 136 | 134 | -2.67 (-2.88, -2.46) | <0.00001 | 0 |  |  |
|  | ≥30 kg/m^2^ | 1 | 29 | 30 | -0.06 (-0.67, 0.55) | 0.85 | - | 62.02 | <0.00001 |
|  | ROB judgement |  |  |  |  |  |  |  |  |
|  | Low | 1 | 37 | 37 | -2.26 (-6.07, 1.55) | 0.25 | - |  |  |
|  | Unclear | 2 | 128 | 127 | -1.38 (-3.94, 1.18) | 0.29 | 98 | 0.14 | 0.71 |
|  | Jadad scale |  |  |  |  |  |  |  |  |
|  | High | 3 | 165 | 164 | -1.55 (-3.80, 0.71) | 0.18 | 97 | - | - |
|  | Assignment |  |  |  |  |  |  |  |  |
|  | Crossover | 1 | 37 | 37 | -2.26 (-6.07, 1.55) | 0.25 | - |  |  |
|  | Parallel | 2 | 128 | 127 | -1.38 (-3.94, 1.18) | 0.29 | 98 | 0.14 | 0.71 |
| Body fat percentage (%) | RS type |  |  |  |  |  |  |  |  |
|  | RS2 | 4 | 189 | 187 | -1.03 (-2.59, 0.54) | 0.20 | 95 |  |  |
|  | RS3 | 1 | 12 | 13 | -1.40 (-4.80, 2.00) | 0.42 | - | 0.04 | 0.85 |
|  | Delivery mode |  |  |  |  |  |  |  |  |
|  | Supplement | 4 | 176 | 175 | -1.88 (-2.10, -1.67) | <0.00001 | 0 |  |  |
|  | Food | 1 | 25 | 25 | -0.14 (-0.61, 0.33) | 0.56 | - | 43.59 | <0.00001 |
|  | Dose |  |  |  |  |  |  |  |  |
|  | <30 g/day | 3 | 65 | 66 | -0.16 (-0.63, 0.30) | 0.49 | 0 |  |  |

**Supplementary Table 5.** Cont.

| Effect size | Subgroups | Trial count | Intervention group size | Control group size | MD (95% CI) | P for overall effect | I^2^ in subgroup (%) | Chi-square | P for subgroup differences |
| --- | --- | --- | --- | --- | --- | --- | --- | --- | --- |
| Body fat percentage (%) | ≥30 g/day | 2 | 136 | 134 | -1.89 (-2.10, -1.67) | <0.00001 | 0 | 43.15 | <0.00001 |
|  | Duration |  |  |  |  |  |  |  |  |
|  | <8 weeks | 3 | 65 | 66 | -0.16 (-0.63, 0.30) | 0.49 | 0 |  |  |
|  | ≥8 weeks | 2 | 136 | 134 | -1.89 (-2.10, -1.67) | <0.00001 | 0 | 43.15 | <0.00001 |
|  | Region |  |  |  |  |  |  |  |  |
|  | Western developed | 1 | 28 | 28 | - | - | - |  |  |
|  | Others | 4 | 173 | 172 | -1.07 (-2.51, 0.36) | 0.14 | 93 | - | - |
|  | Disease |  |  |  |  |  |  |  |  |
|  | Overweight/obesity | 3 | 74 | 75 | -0.18 (-0.64, 0.29) | 0.45 | 0 |  |  |
|  | T2DM | 1 | 28 | 28 | - | - | - |  |  |
|  | NAFLD | 1 | 99 | 97 | -1.89 (-2.10, -1.68) | <0.00001 | - | 43.15 | <0.00001 |
|  | Mean age |  |  |  |  |  |  |  |  |
|  | <45 years | 3 | 148 | 147 | -1.88 (-2.10, -1.67) | <0.00001 | 0 |  |  |
|  | ≥45 years | 2 | 53 | 53 | -0.14 (-0.61, 0.33) | 0.56 | - | 43.59 | <0.00001 |
|  | Mean BMI |  |  |  |  |  |  |  |  |
|  | <30 kg/m^2^ | 3 | 148 | 147 | -1.88 (-2.10, -1.67) | <0.00001 | 0 |  |  |
|  | ≥30 kg/m^2^ | 2 | 53 | 53 | -0.14 (-0.61, 0.33) | 0.56 | - | 43.59 | <0.00001 |
|  | ROB judgement |  |  |  |  |  |  |  |  |
|  | Low | 1 | 37 | 37 | -1.03 (-4.60, 2.54) | 0.57 | - |  |  |
|  | Unclear | 4 | 164 | 163 | -1.08 (-2.64, 0.47) | 0.17 | 95 | 0.00 | 0.98 |
|  | Jadad scale |  |  |  |  |  |  |  |  |
|  | Low | 2 | 53 | 53 | -0.14 (-0.61, 0.33) | 0.56 | - |  |  |
|  | High | 3 | 148 | 147 | -1.88 (-2.10, -1.67) | <0.00001 | 0 | 43.59 | <0.00001 |
|  | Assignment |  |  |  |  |  |  |  |  |
|  | Crossover | 3 | 77 | 78 | -1.22 (-3.69, 1.24) | 0.33 | 0 |  |  |
|  | Parallel | 2 | 124 | 122 | -1.03 (-2.74, 0.69) | 0.24 | 98 | 0.02 | 0.90 |
| SBP (mmHg) | RS type |  |  |  |  |  |  |  |  |
|  | RS2 | 6 | 267 | 255 | -0.66 (-3.35, 2.03) | 0.63 | 58 |  |  |
|  | RS3 | 2 | 39 | 40 | -2.99 (-22.19, 16.21) | 0.76 | 76 | 0.06 | 0.81 |

**Supplementary Table 5.** Cont.

| Effect size | Subgroups | Trial count | Intervention group size | Control group size | MD (95% CI) | P for overall effect | I^2^ in subgroup (%) | Chi-square | P for subgroup differences |
| --- | --- | --- | --- | --- | --- | --- | --- | --- | --- |
| SBP (mmHg) | Delivery mode |  |  |  |  |  |  |  |  |
|  | Supplement | 5 | 198 | 198 | -3.21 (-4.00, -2.42) | <0.00001 | 3 |  |  |
|  | Food | 3 | 108 | 97 | 3.59 (0.39, 6.79) | 0.03 | 0 | 16.35 | <0.0001 |
|  | Dose |  |  |  |  |  |  |  |  |
|  | <30 g/day | 6 | 170 | 161 | 1.42 (-1.61, 4.45) | 0.36 | 24 |  |  |
|  | ≥30 g/day | 2 | 136 | 134 | -2.73 (-5.16, -0.30) | 0.03 | 39 | 4.38 | 0.04 |
|  | Duration |  |  |  |  |  |  |  |  |
|  | <8 weeks | 3 | 58 | 59 | 1.75 (-5.00, 8.50) | 0.61 | 53 |  |  |
|  | ≥8 weeks | 5 | 248 | 236 | -1.20 (-3.85, 1.45) | 0.37 | 54 | 0.63 | 0.43 |
|  | Region |  |  |  |  |  |  |  |  |
|  | Western developed | 3 | 75 | 76 | -0.72 (-5.17, 3.73) | 0.75 | 68 |  |  |
|  | Others | 4 | 231 | 219 | -2.11 (-3.95, -0.27) | 0.02 | 53 | 0.32 | 0.57 |
|  | Disease |  |  |  |  |  |  |  |  |
|  | Overweight/obesity | 3 | 70 | 71 | -1.22 (-5.66, 3.22) | 0.59 | 14 |  |  |
|  | MetS | 2 | 46 | 46 | 3.99 (-0.16, 8.13) | 0.06 | 0 |  |  |
|  | Prediabetes/T2DM | 2 | 91 | 81 | 0.94 (-3.50, 5.38) | 0.68 | 27 |  |  |
|  | NAFLD | 1 | 99 | 97 | -3.34 (-3.62, -3.06) | <0.00001 | - | 16.28 | 0.001 |
|  | Mean age |  |  |  |  |  |  |  |  |
|  | <45 years | 5 | 196 | 195 | -0.90 (-4.79, 2.99) | 0.65 | 68 |  |  |
|  | ≥45 years | 3 | 110 | 100 | 1.43 (-1.83, 4.68) | 0.39 | 0 | 0.81 | 0.37 |
|  | Mean BMI |  |  |  |  |  |  |  |  |
|  | <30 kg/m^2^ | 3 | 148 | 147 | -2.84 (-5.86, 0.18) | 0.07 | 37 |  |  |
|  | ≥30 kg/m^2^ | 4 | 139 | 129 | 1.76 (-1.25, 4.77) | 0.25 | 11 | 4.47 | 0.03 |
|  | ROB judgement |  |  |  |  |  |  |  |  |
|  | Low | 1 | 37 | 37 | -0.22 (-5.00, 4.56) | 0.93 | - |  |  |
|  | Unclear | 4 | 159 | 159 | -2.22 (-5.55, 1.11) | 0.19 | 43 |  |  |
|  | High | 3 | 110 | 99 | 2.88 (-0.38, 6.13) | 0.08 | 0 | 4.66 | 0.10 |
|  | Jadad scale |  |  |  |  |  |  |  |  |
|  | Low | 2 | 89 | 78 | 0.09 (-2.01, 2.19) | 0.93 | 0 |  |  |

**Supplementary Table 5.** Cont.

| Effect size | Subgroups | Trial count | Intervention group size | Control group size | MD (95% CI) | P for overall effect | I^2^ in subgroup (%) | Chi-square | P for subgroup differences |
| --- | --- | --- | --- | --- | --- | --- | --- | --- | --- |
| SBP (mmHg) | High | 6 | 217 | 217 | -2.29 (-4.30, -0.29) | 0.02 | 49 | 2.60 | 0.11 |
|  | Assignment |  |  |  |  |  |  |  |  |
|  | Crossover | 5 | 116 | 117 | 1.13 (-2.38, 4.64) | 0.53 | 29 |  |  |
|  | Parallel | 3 | 190 | 178 | -1.18 (-5.13, 2.76) | 0.56 | 69 | 0.74 | 0.39 |
| DBP (mmHg) | RS type |  |  |  |  |  |  |  |  |
|  | RS2 | 6 | 267 | 255 | -1.94 (-3.96, 0.07) | 0.06 | 68 |  |  |
|  | RS3 | 2 | 39 | 40 | 1.05 (-2.62, 4.71) | 0.57 | 0 | 1.97 | 0.16 |
|  | Delivery mode |  |  |  |  |  |  |  |  |
|  | Supplement | 5 | 198 | 198 | -3.51 (-3.71, -3.32) | <0.00001 | 0 |  |  |
|  | Food | 3 | 108 | 97 | 0.43 (-1.41, 2.28) | 0.64 | 0 | 17.39 | <0.0001 |
|  | Dose |  |  |  |  |  |  |  |  |
|  | <30 g/day | 6 | 170 | 161 | -0.53 (-2.55, 1.48) | 0.60 | 24 |  |  |
|  | ≥30 g/day | 2 | 136 | 134 | -3.30 (-4.50, -2.10) | <0.00001 | 22 | 5.34 | 0.02 |
|  | Duration |  |  |  |  |  |  |  |  |
|  | <8 weeks | 3 | 58 | 59 | 1.31 (-1.35, 3.97) | 0.33 | 0 |  |  |
|  | ≥8 weeks | 5 | 248 | 236 | -2.57 (-4.35, -0.78) | 0.005 | 55 | 5.63 | 0.02 |
|  | Region |  |  |  |  |  |  |  |  |
|  | Western developed | 3 | 75 | 76 | -0.72 (-5.17, 3.73) | 0.75 | 68 |  |  |
|  | Others | 5 | 231 | 219 | -2.11 (-3.95, -0.27) | 0.02 | 53 | 0.32 | 0.57 |
|  | Disease |  |  |  |  |  |  |  |  |
|  | Overweight or obesity | 3 | 70 | 71 | -1.53 (-4.26, 1.20) | 0.27 | 0 |  |  |
|  | MetS | 2 | 46 | 46 | -0.72 (-5.17, 3.73) | 0.75 | 68 |  |  |
|  | Prediabetes/T2DM | 2 | 91 | 81 | -0.36 (-2.79, 2.07) | 0.77 | - |  |  |
|  | NAFLD | 1 | 99 | 97 | -3.52 (-3.72, -3.32) | <0.00001 | - | 9.91 | 0.02 |
|  | Mean age |  |  |  |  |  |  |  |  |
|  | <45 years | 5 | 196 | 195 | -1.96 (-4.05, 0.12) | 0.06 | 47 |  |  |
|  | ≥45 years | 3 | 110 | 100 | -1.13 (-4.67, 2.41) | 0.53 | 64 | 0.16 | 0.69 |
|  | Mean BMI |  |  |  |  |  |  |  |  |

**Supplementary Table 5.** Cont.

| Effect size | Subgroups | Trial count | Intervention group size | Control group size | MD (95% CI) | P for overall effect | I^2^ in subgroup (%) | Chi-square | P for subgroup differences |
| --- | --- | --- | --- | --- | --- | --- | --- | --- | --- |
| DBP (mmHg) | <30 kg/m^2^ | 3 | 148 | 147 | -3.51 (-3.71, -3.31) | <0.00001 | 0 |  |  |
|  | ≥30 kg/m^2^ | 4 | 139 | 129 | -1.27 (-4.02, 1.48) | 0.37 | 43 | 2.53 | 0.11 |
|  | ROB judgement |  |  |  |  |  |  |  |  |
|  | Low | 1 | 37 | 37 | -1.59 (-4.92, 1.74) | 0.35 | - |  |  |
|  | Unclear | 4 | 159 | 159 | -2.31 (-5.39, 0.77) | 0.14 | 64 |  |  |
|  | High | 3 | 110 | 99 | -0.15 (-2.13, 1.83) | 0.88 | 0 | 1.52 | 0.47 |
|  | Jadad scale |  |  |  |  |  |  |  |  |
|  | Low | 2 | 89 | 78 | 0.09 (-2.01, 2.19) | 0.93 | 0 |  |  |
|  | High | 6 | 217 | 217 | -2.29 (-4.30, -0.29) | 0.02 | 49 | 2.60 | 0.11 |
|  | Assignment |  |  |  |  |  |  |  |  |
|  | Crossover | 5 | 116 | 117 | -0.06 (-2.03, 1.90) | 0.95 | 0 |  |  |
|  | Parallel | 3 | 190 | 178 | -2.85 (-5.38, -0.31) | 0.03 | 73 | 2.88 | 0.09 |

Abbreviation: BW, body weight; BMI, body mass index; WC, waist circumference; FM, fat mass; DBP, diastolic blood pressure; SBP, systolic blood pressure; MetS, metabolic syndrome; NAFLD, non-alcoholic fatty liver disease; T2DM, type 2 diabetes; CI, confidential intervals; MD, mean difference; ROB, risk of bias; RS, resistant starch.

**Supplementary Table 6.** Subgroup Analysis of Glycemic Profiles

| Effect size | Subgroups | Trial count | Intervention group size | Control group size | MD (95% CI) | P for overall effect | I^2^ in subgroup (%) | Chi-square | P for subgroup differences |
| --- | --- | --- | --- | --- | --- | --- | --- | --- | --- |
| FINS (μU/mL) | RS type |  |  |  |  |  |  |  |  |
|  | RS2 | 10 | 366 | 357 | -0.61 (-0.93, -0.28) | 0.0003 | 60 |  |  |
|  | RS3 | 2 | 39 | 40 | -0.46 (-3.70, 2.78) | 0.78 | 50 | 0.01 | 0.93 |
|  | Delivery mode |  |  |  |  |  |  |  |  |
|  | Supplement | 6 | 217 | 217 | -2.99 (-3.23, -2.74) | <0.00001 | 0 |  |  |
|  | Food | 6 | 188 | 180 | -2.05 (-4.61, 0.51) | 0.12 | 85 | 0.51 | 0.47 |
|  | Dose |  |  |  |  |  |  |  |  |
|  | <30 g/day | 11 | 306 | 300 | -2.01 (-3.63, -0.39) | 0.02 | 79 |  |  |
|  | ≥30 g/day | 1 | 99 | 97 | -3.03 (-3.28, -2.78) | <0.00001 | 82 | 1.49 | 0.22 |
|  | Duration |  |  |  |  |  |  |  |  |
|  | <8 weeks | 7 | 166 | 170 | -2.20 (-4.42, 0.02) | 0.05 | 84 |  |  |
|  | ≥8 weeks | 5 | 239 | 227 | -2.74 (-3.48, -1.99) | <0.00001 | 14 | 0.20 | 0.65 |
|  | Region |  |  |  |  |  |  |  |  |
|  | Western developed | 6 | 114 | 115 | -2.12 (-5.32, 1.09) | 0.20 | 83 |  |  |
|  | Others | 1 | 291 | 282 | -2.27 (-3.12, -1.43) | <0.00001 | - | 0.01 | 0.93 |
|  | Disease |  |  |  |  |  |  |  |  |
|  | Overweight/obesity | 3 | 57 | 58 | -3.56 (-6.05, -1.06) | 0.005 | 60 |  |  |
|  | MetS | 2 | 46 | 46 | -0.82 (-9.17, 7.52) | 0.85 | 55 |  |  |
|  | Prediabetes/T2DM | 6 | 203 | 196 | -1.53 (-2.45, -0.60) | 0.001 | 0 |  |  |
|  | NAFLD | 1 | 99 | 97 | -4.99 (-5.44, -4.54) | <0.00001 | - | 44.57 | <0.00001 |
|  | Mean age |  |  |  |  |  |  |  |  |
|  | <45 years | 5 | 174 | 173 | -2.58 (-3.79, -1.38) | <0.0001 | 13 |  |  |
|  | ≥45 years | 7 | 231 | 224 | -2.40 (-4.26, -0.55) | 0.01 | 85 | 0.03 | 0.87 |
|  | Mean BMI |  |  |  |  |  |  |  |  |
|  | <30 kg/m^2^ | 3 | 152 | 154 | -2.42 (-3.67, -1.16) | 0.0002 | 60 |  |  |
|  | ≥30 kg/m^2^ | 8 | 234 | 224 | -1.86 (-3.86, 0.14) | 0.07 | 79 | 0.21 | 0.65 |
|  | ROB judgement |  |  |  |  |  |  |  |  |
|  | Low | 1 | 28 | 28 | -3.08 (-5.32, -0.84) | 0.007 | - |  |  |
|  | Unclear | 8 | 267 | 270 | -2.60 (-3.87, -1.33) | <0.0001 | 88 |  |  |

**Supplementary Table 6.** Cont.

| Effect size | Subgroups | Trial count | Intervention group size | Control group size | MD/SMD (95% CI) | P for overall effect | I^2^ in subgroup (%) | Chi-square | P for subgroup differences |
| --- | --- | --- | --- | --- | --- | --- | --- | --- | --- |
| FINS (μU/mL) | High | 3 | 110 | 99 | (-0.81, -3.04, 1.43) | 0.48 | 0 | 2.38 | 0.30 |
|  | Jadad scale |  |  |  |  |  |  |  |  |
|  | Low | 3 | 117 | 106 | -1.05 (-2.74, 0.64) | 0.22 | 0 |  |  |
|  | High | 9 | 288 | 291 | -2.76 (-3.95, -1.56) | <0.00001 | 85 | 2.61 | 0.11 |
|  | Assignment |  |  |  |  |  |  |  |  |
|  | Crossover | 7 | 146 | 147 | -2.30 (-4.56, -0.04) | 0.05 | 72 |  |  |
|  | Parallel | 5 | 259 | 250 | -2.22 (-3.29, -1.15) | <0.00001 | 55 | <0.01 | 0.95 |
| HOMA-IR | RS type |  |  |  |  |  |  |  |  |
|  | RS2 | 9 | 337 | 327 | -0.60 (-0.94, -0.26) | 0.0005 | 65 |  |  |
|  | RS3 | 1 | 27 | 27 | 1.00 (-1.85, 3.85) | 0.49 | - | 1.20 | 0.27 |
|  | Delivery mode |  |  |  |  |  |  |  |  |
|  | Supplement | 4 | 176 | 174 | -0.54 (-1.20, 0.13) | 0.11 | 62 |  |  |
|  | Food | 6 | 188 | 180 | -0.46 (-1.09, 0.17) | 0.15 | 69 | 0.03 | 0.87 |
|  | Dose |  |  |  |  |  |  |  |  |
|  | <30 g/day | 9 | 265 | 257 | -0.44 (-0.97, 0.09) | 0.10 | 67 |  |  |
|  | ≥30 g/day | 1 | 99 | 97 | -0.69 (-0.75, -0.63) | <0.00001 | - | 0.83 | 0.36 |
|  | Duration |  |  |  |  |  |  |  |  |
|  | <8 weeks | 2 | 154 | 157 | -0.32 (-1.01, 0.37) | 0.36 | 75 |  |  |
|  | ≥8 weeks | 4 | 210 | 197 | -0.67 (-1.09, -0.24) | 0.002 | 29 | 0.70 | 0.40 |
|  | Region |  |  |  |  |  |  |  |  |
|  | Western developed | 4 | 85 | 85 | -0.45 (-1.52, 0.63) | 0.41 | 60 |  |  |
|  | Others | 6 | 279 | 269 | -0.49 (-0.84, -0.14) | 0.006 | 57 | 0.01 | 0.94 |
|  | Disease |  |  |  |  |  |  |  |  |
|  | Overweight/obesity | 2 | 45 | 45 | -0.95 (-1.92, 0.03) | 0.06 | 66 |  |  |
|  | MetS | 2 | 46 | 46 | -0.06 (-1.27, 1.15) | 0.93 | 0 |  |  |
|  | Prediabetes/T2DM | 5 | 174 | 166 | -0.28 (-0.93, 0.36) | 0.39 | 52 |  |  |
|  | NAFLD | 1 | 99 | 97 | -1.21 (-1.42, -1.00) | <0.00001 | - | 10.19 | 0.02 |
|  | Mean age |  |  |  |  |  |  |  |  |
|  | <45 years | 4 | 162 | 160 | -0.59 (-0.94, -0.25) | 0.0008 | 10 |  |  |

**Supplementary Table 6.** Cont.

| Effect size | Subgroups | Trial count | Intervention group size | Control group size | MD/SMD (95% CI) | P for overall effect | I^2^ in subgroup (%) | Chi-square | P for subgroup differences |
| --- | --- | --- | --- | --- | --- | --- | --- | --- | --- |
| HOMA-IR | ≥45 years | 6 | 202 | 194 | -0.58 (-1.19, 0.04) | 0.07 | 76 | 0.00 | 0.97 |
|  | Mean BMI |  |  |  |  |  |  |  |  |
|  | <30 kg/m^2^ | 2 | 140 | 141 | -0.61 (-0.86, -0.35) | <0.00001 | 57 |  |  |
|  | ≥30 kg/m^2^ | 7 | 205 | 194 | -0.40 (-1.16, 0.37) | 0.31 | 69 | 0.26 | 0.61 |
|  | ROB judgement |  |  |  |  |  |  |  |  |
|  | Low | 1 | 28 | 28 | -1.61 (-2.77, -0.45) | 0.007 | - |  |  |
|  | Unclear | 6 | 226 | 227 | -0.59 (-0.97, -0.21) | 0.003 | 73 |  |  |
|  | High | 3 | 110 | 99 | -0.07 (-0.85, 0.70) | 0.86 | 0 | 4.69 | 0.10 |
|  | Jadad scale |  |  |  |  |  |  |  |  |
|  | Low | 3 | 117 | 106 | 0.35 (-0.42, 1.12) | 0.37 | 0 |  |  |
|  | High | 7 | 247 | 248 | -0.74 (-1.07, -0.41) | <0.0001 | 63 | 6.51 | 0.01 |
|  | Assignment |  |  |  |  |  |  |  |  |
|  | Crossover | 6 | 134 | 134 | -0.21 (-1.09, 0.66) | 0.63 | 70 |  |  |
|  | Parallel | 4 | 230 | 220 | -0.61 (-0.92, -0.31) | <0.0001 | 51 | 0.72 | 0.40 |
| HOMA-β^*^ | RS type |  |  |  |  |  |  |  |  |
|  | RS2 | 3 | 49 | 49 | -1.08 (-2.76, 0.60) | 0.21 | 92 | - | - |
|  | Delivery mode |  |  |  |  |  |  |  |  |
|  | Supplement | 1 | 10 | 10 | -0.57 (-1.47, 0.33) | 0.21 | - |  |  |
|  | Food | 2 | 39 | 39 | -1.33 (-4.02, 1.36) | 0.33 | 96 | 0.27 | 0.60 |
|  | Dose |  |  |  |  |  |  |  |  |
|  | <30 g/day | 2 | 39 | 39 | -1.33 (-4.02, 1.36) | 0.33 | 96 |  |  |
|  | ≥30 g/day | 1 | 10 | 10 | -0.57 (-1.47, 0.33) | 0.21 | - | 0.27 | 0.60 |
|  | Duration |  |  |  |  |  |  |  |  |
|  | <8 weeks | 2 | 39 | 39 | -1.33 (-4.02, 1.36) | 0.33 | 96 |  |  |
|  | ≥8 weeks | 1 | 10 | 10 | -0.57 (-1.47, 0.33) | 0.21 | - | 0.27 | 0.60 |
|  | Region |  |  |  |  |  |  |  |  |
|  | Western developed | 3 | 49 | 49 | -1.08 (-2.76, 0.60) | 0.21 | 92 | - | - |
|  | Disease |  |  |  |  |  |  |  |  |
|  | Overweight/obesity | 1 | 24 | 24 | -2.71 (-3.51, -1.91) | <0.00001 | - |  |  |

**Supplementary Table 6.** Cont.

| Effect size | Subgroups | Trial count | Intervention group size | Control group size | MD/SMD (95% CI) | P for overall effect | I^2^ in subgroup (%) | Chi-square | P for subgroup differences |
| --- | --- | --- | --- | --- | --- | --- | --- | --- | --- |
| HOMA-β^*^ | Insulin resistance | 1 | 10 | 10 | -0.57 (-1.47, 0.33) | 0.21 | - |  |  |
|  | T2DM | 1 | 15 | 15 | 0.04 (-0.68, 0.75) | 0.92 | - | 26.43 | <0.00001 |
|  | Mean age |  |  |  |  |  |  |  |  |
|  | <45 years | 1 | 15 | 15 | 0.04 (-0.68, 0.75) | 0.92 | - |  |  |
|  | ≥45 years | 2 | 34 | 34 | -1.65 (-3.74, 0.44) | 0.12 | 92 | 2.23 | 0.13 |
|  | Mean BMI |  |  |  |  |  |  |  |  |
|  | ≥30 kg/m^2^ | 3 | 49 | 49 | -1.08 (-2.76, 0.60) | 0.21 | 92 | - | - |
|  | ROB judgement |  |  |  |  |  |  |  |  |
|  | Unclear | 3 | 49 | 49 | -1.08 (-2.76, 0.60) | 0.21 | 92 | - | - |
|  | Jadad scale |  |  |  |  |  |  |  |  |
|  | Low | 1 | 10 | 10 | -0.57 (-1.47, 0.33) | 0.21 | - |  |  |
|  | High | 2 | 39 | 39 | -1.33 (-4.02, 1.36) | 0.33 | 96 | 0.27 | 0.60 |
|  | Assignment |  |  |  |  |  |  |  |  |
|  | Crossover | 2 | 39 | 39 | -1.33 (-4.02, 1.36) | 0.33 | 96 |  |  |
|  | Parallel | 1 | 10 | 10 | -0.57 (-1.47, 0.33) | 0.21 | - | 0.27 | 0.60 |

*Effect measure was using standard mean difference. Abbreviation: FINS, fasting insulin; HOMA-IR, homeostatic model assessment of insulin resistance; HOMA-β, homeostatic model assessment of beta-cell function; MetS, metabolic syndrome; NAFLD, non-alcoholic fatty liver disease; T2DM, type 2 diabetes; CI, confidential intervals; MD, mean difference; SMD, standard mean difference; ROB, risk of bias; RS, resistant starch.

**Supplementary Table 7.** Subgroup Analysis of Lipid Profiles

| Effect size | Subgroups | Trial count | Intervention group size | Control group size | MD/SMD (95% CI) | P for overall effect | I^2^ in subgroup (%) | Chi-square | P for subgroup differences |
| --- | --- | --- | --- | --- | --- | --- | --- | --- | --- |
| TG (mmol/L) | RS type |  |  |  |  |  |  |  |  |
|  | RS2 | 10 | 382 | 376 | -0.13 (-0.35, 0.10) | 0.28 | 90 |  |  |
|  | RS3 | 2 | 50 | 48 | 0.09 (-0.38, 0.56) | 0.70 | 0 | 0.68 | 0.41 |
|  | Delivery mode |  |  |  |  |  |  |  |  |
|  | Supplement | 9 | 326 | 329 | 0.10 (-0.37, 0.17) | 0.46 | 89 |  |  |
|  | Food | 3 | 106 | 95 | -0.14 (-0.32, 0.04) | 0.12 | 0 | 0.06 | 0.80 |
|  | Dose |  |  |  |  |  |  |  |  |
|  | <30 g/day | 10 | 296 | 290 | -0.07 (-0.20, 0.06) | 0.29 | 25 |  |  |
|  | ≥30 g/day | 2 | 136 | 134 | -0.34 (-0.81, 0.13) | 0.16 | 92 | 1.16 | 0.28 |
|  | Duration |  |  |  |  |  |  |  |  |
|  | <8 weeks | 4 | 84 | 85 | 0.12 (-0.21, 0.46) | 0.47 | 44 |  |  |
|  | ≥8 weeks | 8 | 348 | 339 | -0.19 (-0.42, 0.03) | 0.09 | 88 | 2.37 | 0.12 |
|  | Region |  |  |  |  |  |  |  |  |
|  | Western developed | 2 | 48 | 49 | -0.09 (-0.33, 0.16) | 0.49 | 0 |  |  |
|  | Others | 10 | 384 | 375 | -0.11 (-0.35, 0.13) | 0.37 | 89 | 0.02 | 0.89 |
|  | Disease |  |  |  |  |  |  |  |  |
|  | Overweight/obesity | 4 | 95 | 96 | -0.06 (-0.24, 0.13) | 0.55 | 0 |  |  |
|  | MetS | 1 | 19 | 19 | -0.10 (-0.55, 0.35) | 0.66 | - |  |  |
|  | Prediabetes/T2DM | 5 | 181 | 177 | -0.08 (-0.30, 0.15) | 0.49 | 64 |  |  |
|  | Hyperlipidemia | 1 | 38 | 35 | 0.10 (-0.39, 0.59) | 0.69 | - |  |  |
|  | NAFLD | 1 | 99 | 97 | -0.56 (-0.59, -0.53) | <0.00001 | - | 55.45 | <0.00001 |
|  | Mean age |  |  |  |  |  |  |  |  |
|  | <45 years | 4 | 169 | 168 | 0.24 (-0.62， 0.14) | 0.22 | 85 |  |  |
|  | ≥45 years | 8 | 263 | 256 | -0.07 (-0.22, 0.09) | 0.39 | 41 | 0.67 | 0.41 |
|  | Mean BMI |  |  |  |  |  |  |  |  |
|  | <30 kg/m^2 | 5 | 220 | 218 | -0.20 (-0.53, 0.13) | 0.24 | 88 |  |  |
|  | ≥30 kg/m^2 | 6 | 193 | 187 | -0.05 (-0.26, 0.16) | 0.62 | 55 | 0.55 | 0.46 |
|  | ROB judgement |  |  |  |  |  |  |  |  |
|  | Low | 2 | 65 | 69 | -0.18 (-0.47, 0.10) | 0.21 | 30 |  |  |

**Supplementary Table 7.** Cont.

| Effect size | Subgroups | Trial count | Intervention group size | Control group size | MD (95% CI) | P for overall effect | I^2^ in subgroup (%) | Chi-square | P for subgroup differences |
| --- | --- | --- | --- | --- | --- | --- | --- | --- | --- |
| TG (mmol/L) | Unclear | 7 | 250 | 247 | -0.04 (-0.41, 0.32) | 0.82 | 89 |  |  |
|  | High | 3 | 117 | 108 | -0.15 (-0.30, 0.00) | 0.04 | 0 | 0.37 | 0.83 |
|  | Jadad scale |  |  |  |  |  |  |  |  |
|  | Low | 4 | 149 | 140 | -0.01 (-0.27, 0.25) | 0.95 | 68 |  |  |
|  | High | 8 | 283 | 284 | -0.18 (-0.44, 0.08) | 0.17 | 83 | 0.85 | 0.36 |
|  | Assignment |  |  |  |  |  |  |  |  |
|  | Crossover | 5 | 261 | 251 | -0.27 (-0.54, 0.00) | 0.05 | 86 |  |  |
|  | Parallel | 7 | 286 | 276 | -0.17 (-0.45, 0.10) | 0.22 | 87 | 0.25 | 0.62 |
| HDL-C (mmol/L) | RS type |  |  |  |  |  |  |  |  |
|  | RS2 | 10 | 382 | 376 | 0.03 (-0.02, 0.07) | 0.30 | 71 |  |  |
|  | RS3 | 2 | 50 | 48 | 0.00 (-0.10, 0.11) | 0.94 | 0 | 0.13 | 0.72 |
|  | Delivery mode |  |  |  |  |  |  |  |  |
|  | Supplement | 9 | 326 | 329 | 0.03 (-0.02, 0.08) | 0.27 | 73 |  |  |
|  | Food | 3 | 106 | 95 | 0.00 (-0.10, 0.09) | 0.92 | 0 | 0.35 | 0.55 |
|  | Dose |  |  |  |  |  |  |  |  |
|  | <30 g/day | 10 | 296 | 290 | 0.02 (-0.04, 0.08) | 0.52 | 59 |  |  |
|  | ≥30 g/day | 2 | 136 | 134 | 0.03 (-0.06, 0.13) | 0.46 | 70 | 0.09 | 0.77 |
|  | Duration |  |  |  |  |  |  |  |  |
|  | <8 weeks | 4 | 84 | 85 | -0.02 (-0.08, 0.05) | 0.65 | 0 |  |  |
|  | ≥8 weeks | 8 | 348 | 339 | 0.03 (-0.02, 0.09) | 0.20 | 73 | 1.32 | 0.25 |
|  | Region |  |  |  |  |  |  |  |  |
|  | Western developed | 2 | 48 | 49 | 0.05 (-0.03, 0.13) | 0.21 | 17 |  |  |
|  | Others | 10 | 384 | 375 | 0.02 (-0.03, 0.07) | 0.49 | 71 | 0.41 | 0.52 |
|  | Disease |  |  |  |  |  |  |  |  |
|  | Overweight/obesity | 4 | 95 | 96 | -0.01 (-0.07, 0.05) | 0.76 | 0 |  |  |
|  | MetS | 1 | 48 | 49 | -0.03 (-0.20, 0.14) | 0.72 | - |  |  |
|  | Prediabetes/T2DM | 5 | 152 | 147 | 0.03 (-0.06, 0.13) | 0.50 | 81 |  |  |
|  | Hyperlipidemia | 1 | 38 | 35 | 0.01 (-0.15, 0.17) | 0.90 | - |  |  |

**Supplementary Table 7.** Cont.

| Effect size | Subgroups | Trial count | Intervention group size | Control group size | MD (95% CI) | P for overall effect | I^2^ in subgroup (%) | Chi-square | P for subgroup differences |
| --- | --- | --- | --- | --- | --- | --- | --- | --- | --- |
| HDL-C (mmol/L) | NAFLD | 1 | 99 | 97 | 0.07 (0.06, 0.08) | <0.00001 | - | 9.06 | 0.06 |
|  | Mean age |  |  |  |  |  |  |  |  |
|  | <45 years | 4 | 169 | 168 | 0.07 (0.06, 0.07) | <0.00001 | 54 |  |  |
|  | ≥45 years | 8 | 263 | 256 | 0.03 (-0.01, 0.07) | 0.14 | 67 | 4.20 | 0.04 |
|  | Mean BMI |  |  |  |  |  |  |  |  |
|  | <30 kg/m^2^ | 5 | 220 | 218 | -0.01 (-0.09, 0.08) | 0.90 | 77 |  |  |
|  | ≥30 kg/m^2^ | 6 | 193 | 187 | 0.05 (-0.02, 0.12) | 0.15 | 60 | 1.03 | 0.31 |
|  | ROB judgement |  |  |  |  |  |  |  |  |
|  | Low | 2 | 65 | 69 | 0.09 (-0.15, 0.33) | 0.45 | 90 |  |  |
|  | Unclear | 7 | 250 | 247 | 0.06 (0.03, 0.08) | <0.00001 | 11 |  |  |
|  | High | 3 | 117 | 108 | -0.04 (-0.11, 0.04) | 0.33 | 30 | 6.04 | 0.05 |
|  | Jadad scale |  |  |  |  |  |  |  |  |
|  | Low | 4 | 149 | 140 | -0.04 (-0.10, 0.01) | 0.14 | 0 |  |  |
|  | High | 8 | 283 | 284 | 0.05 (0.01, 0.09) | 0.02 | 53 | 6.59 | 0.01 |
|  | Assignment |  |  |  |  |  |  |  |  |
|  | Crossover | 5 | 117 | 118 | -0.01 (-0.06, 0.03) | 0.56 | 0 |  |  |
|  | Parallel | 7 | 315 | 306 | 0.05 (-0.01, 0.11) | 0.12 | 71 | 2.47 | 0.12 |

Abbreviation: TG, triglycerides; HDL-C, high-density lipoprotein cholesterol; MetS, metabolic syndrome; NAFLD, non-alcoholic fatty liver disease; T2DM, type 2 diabetes; CI, confidential intervals; MD, mean difference; ROB, risk of bias; RS, resistant starch.

**Supplementary Table 8.** Subgroup analysis of Inflammatory Factors

| Effect size | Subgroups | Trial count | Intervention group size | Control group size | MD/SMD (95% CI) | P for overall effect | I^2^ in subgroup (%) | Chi-square | P for subgroup differences |
| --- | --- | --- | --- | --- | --- | --- | --- | --- | --- |
| TNF-α* | RS type |  |  |  |  |  |  |  |  |
|  | RS2 | 6 | 252 | 257 | -0.52 (-1.00, -0.05) | 0.03 | 84 | - | - |
|  | Delivery mode |  |  |  |  |  |  |  |  |
|  | Supplement | 5 | 227 | 232 | -0.51 (-1.07, 0.05) | 0.07 | 87 |  |  |
|  | Food | 1 | 25 | 25 | -0.57 (-1.13, 0.00) | 0.05 | - | 0.02 | 0.89 |
|  | Dose |  |  |  |  |  |  |  |  |
|  | <30 g/day | 4 | 116 | 123 | -0.39 (-0.97, 0.18) | 0.18 | 79 |  |  |
|  | ≥30 g/day | 2 | 136 | 134 | -0.76 (-1.56, 0.04) | 0.06 | 88 | 0.53 | 0.47 |
|  | Duration |  |  |  |  |  |  |  |  |
|  | <8 weeks | 1 | 25 | 25 | -0.57 (-1.13, 0.00) | 0.05 | - |  |  |
|  | ≥8 weeks | 5 | 227 | 232 | -0.51 (-1.07, 0.05) | 0.07 | 87 | 0.02 | 0.89 |
|  | Region |  |  |  |  |  |  |  |  |
|  | Western developed | 1 | 29 | 30 | -0.82 (-1.36, -0.29) | 0.002 | - |  |  |
|  | Others | 5 | 223 | 227 | -0.46 (-1.03, 0.10) | 0.11 | 87 | 0.83 | 0.36 |
|  | Disease |  |  |  |  |  |  |  |  |
|  | Overweight/obesity | 2 | 62 | 62 | -0.42 (-0.78, -0.07) | 0.02 | 0 |  |  |
|  | Prediabetes/T2DM | 3 | 91 | 98 | -0.34 (-1.11, 0.43) | 0.39 | 86 |  |  |
|  | NAFLD | 1 | 99 | 97 | -1.15 (-1.45, -0.84) | <0.00001 | - | 10.69 | 0.005 |
|  | Mean age |  |  |  |  |  |  |  |  |
|  | <45 years | 2 | 136 | 134 | -0.76 (-1.56, 0.04) | 0.06 | 88 |  |  |
|  | ≥45 years | 4 | 116 | 123 | -0.39 (-0.97, 0.18) | 0.18 | 79 | 0.53 | 0.47 |
|  | Mean BMI |  |  |  |  |  |  |  |  |
|  | <30 kg/m^2^ | 3 | 170 | 170 | -0.37 (-1.30, 0.56) | 0.44 | 94 |  |  |
|  | ≥30 kg/m^2^ | 3 | 82 | 87 | -0.68 (-0.99, -0.36) | <0.0001 | 0 | 0.37 | 0.54 |
|  | ROB judgement |  |  |  |  |  |  |  |  |
|  | Low | 2 | 65 | 69 | -0.46 (-0.80, -0.12) | 0.009 | 0 |  |  |
|  | Unclear | 3 | 153 | 152 | -0.91 (-1.26, -0.57) | <0.00001 | 44 |  |  |
|  | High | 1 | 34 | 36 | 0.41 (-0.06, 0.88) | 0.09 | - | 19.45 | <0.0001 |
|  | Jadad scale |  |  |  |  |  |  |  |  |
|  | Low | 2 | 59 | 61 | -0.07 (-1.02, 0.89) | 0.89 | 85 |  |  |

**Supplementary Table 8.** Cont.

| Effect size | Subgroups | Trial count | Intervention group size | Control group size | MD/SMD (95% CI) | P for overall effect | I^2^ in subgroup (%) | Chi-square | P for subgroup differences |
| --- | --- | --- | --- | --- | --- | --- | --- | --- | --- |
| TNF-α* | High | 4 | 193 | 196 | -0.76 (-1.15, -0.36) | 0.0002 | 68 | 1.71 | 0.19 |
|  | Assignment |  |  |  |  |  |  |  |  |
|  | Crossover | 1 | 37 | 37 | -0.33 (-0.79, 0.13) | 0.16 | - |  |  |
|  | Parallel | 5 | 215 | 220 | -0.56 (-1.13, 0.01) | 0.05 | 87 | 0.38 | 0.54 |
| IL-6 (pg/mL) | RS type |  |  |  |  |  |  |  |  |
|  | RS2 | 4 | 198 | 202 | -0.12 (-0.50, 0.26) | 0.54 | 65 | - | - |
|  | Delivery mode |  |  |  |  |  |  |  |  |
|  | Supplement | 4 | 198 | 202 | -0.12 (-0.50, 0.26) | 0.54 | 65 | - | - |
|  | Dose |  |  |  |  |  |  |  |  |
|  | <30 g/day | 2 | 62 | 68 | 0.05 (-2.30, 2.40) | 0.96 | 85 |  |  |
|  | ≥30 g/day | 2 | 136 | 134 | -0.15 (-0.35, 0.05) | 0.13 | 45 | 0.03 | 0.86 |
|  | Duration |  |  |  |  |  |  |  |  |
|  | ≥8 weeks | 4 | 198 | 202 | -0.12 (-0.50, 0.26) | 0.54 | 65 | - | - |
|  | Region |  |  |  |  |  |  |  |  |
|  | Others | 4 | 198 | 202 | -0.12 (-0.50, 0.26) | 0.54 | 65 | - | - |
|  | Disease |  |  |  |  |  |  |  |  |
|  | Overweight/obesity | 1 | 37 | 37 | 0.03 (-0.32, 0.38) | 0.86 | - |  |  |
|  | T2DM | 2 | 62 | 68 | 0.05 (-2.30, 2.40) | 0.96 | 85 |  |  |
|  | NAFLD | 1 | 99 | 97 | -0.21 (-0.26, -0.16) | <0.00001 | - | 1.86 | 0.39 |
|  | Mean age |  |  |  |  |  |  |  |  |
|  | <45 years | 2 | 136 | 134 | -0.15 (-0.35, 0.05) | 0.13 | 45 |  |  |
|  | ≥45 years | 2 | 62 | 68 | 0.05 (-2.30, 2.40) | 0.96 | 85 | 0.03 | 0.86 |
|  | Mean BMI |  |  |  |  |  |  |  |  |
|  | <30 kg/m^2^ | 3 | 170 | 170 | -0.03 (-0.40, 0.33) | 0.87 | 67 |  |  |
|  | ≥30 kg/m^2^ | 1 | 28 | 28 | -1.10 (-2.19, -0.01) | 0.05 | - | 3.30 | 0.07 |
|  | ROB judgement |  |  |  |  |  |  |  |  |
|  | Low | 2 | 65 | 69 | -0.41 (-1.49, 0.67) | 0.46 | 73 |  |  |
|  | Unclear | 1 | 99 | 97 | -0.21 (-0.26, -0.16) | <0.00001 | - |  |  |
|  | High | 3 | 34 | 36 | 1.30 (-0.13, 2.73) | 0.07 | - | 4.42 | 0.11 |
|  | Jadad scale |  |  |  |  |  |  |  |  |

**Supplementary Table 8.** Cont.

| Effect size | Subgroups | Trial count | Intervention group size | Control group size | MD/SMD (95% CI) | P for overall effect | I^2^ in subgroup (%) | Chi-square | P for subgroup differences |
| --- | --- | --- | --- | --- | --- | --- | --- | --- | --- |
| IL-6 (pg/mL) | Low | 1 | 34 | 36 | 1.30 (-0.13, 2.73) | 0.07 | - |  |  |
|  | High | 3 | 164 | 166 | -0.19 (-0.47, 0.10) | 0.20 | 54 | 3.99 | 0.05 |
|  | Assignment |  |  |  |  |  |  |  |  |
|  | Crossover | 1 | 37 | 37 | 0.03 (-0.32, 0.38) | 0.86 | - |  |  |
|  | Parallel | 3 | 161 | 165 | -0.13 (-1.06, 0.81) | 0.79 | 71 | 0.09 | 0.76 |

*Effect measure was using standard mean difference. Abbreviation: TNF-α, tumor necrosis factor-alpha; IL-6, interleukin-6; NAFLD, non-alcoholic fatty liver disease; T2DM, type 2 diabetes; CI, confidential intervals; MD, mean difference; SMD, standard mean difference; ROB, risk of bias; RS, resistant starch.

**Supplementary Table 9.** Subgroup analysis of Oxidative Stress Biomarkers

| Effect size | Subgroups | Trial count | Intervention group size | Control group size | SMD (95% CI) | P for overall effect | I^2^ in subgroup (%) | Chi-square | P for subgroup differences |
| --- | --- | --- | --- | --- | --- | --- | --- | --- | --- |
| MDA* | RS type |  |  |  |  |  |  |  |  |
|  | RS2 | 4 | 124 | 129 | -0.34 (-0.70, 0.03) | 0.07 | 52 |  |  |
|  | RS3 | 2 | 65 | 62 | -0.22 (-1.24, 0.79) | 0.67 | 88 | 0.04 | 0.84 |
|  | Delivery mode |  |  |  |  |  |  |  |  |
|  | Supplement | 4 | 121 | 120 | -0.36 (-0.76, 0.04) | 0.08 | 59 |  |  |
|  | Food | 2 | 68 | 71 | -0.17 (-1.08, 0.73) | 0.71 | 85 | 0.13 | 0.71 |
|  | Dose |  |  |  |  |  |  |  |  |
|  | <30 g/day | 6 | 189 | 191 | -0.30 (-0.65, 0.05) | 0.09 | 65 | - | - |
|  | Duration |  |  |  |  |  |  |  |  |
|  | <8 weeks | 2 | 68 | 71 | -0.17 (-1.08, 0.73) | 0.71 | 85 |  |  |
|  | ≥8 weeks | 4 | 121 | 120 | -0.36 (-0.76, 0.04) | 0.08 | 59 | 0.13 | 0.71 |
|  | Region |  |  |  |  |  |  |  |  |
|  | Western developed | 1 | 27 | 27 | 0.30 (-0.23, 0.84) | 0.27 | - |  |  |
|  | Others | 5 | 162 | 164 | -0.42 (-0.74, -0.10) | 0.01 | 52 | 5.14 | 0.02 |
|  | Disease |  |  |  |  |  |  |  |  |
|  | Overweight/obesity | 1 | 21 | 21 | 0.00 (-0.60, 0.60) | 1.00 | - |  |  |
|  | MetS | 1 | 27 | 27 | 0.30 (-0.23, 0.84) | 0.27 | - |  |  |
|  | T2DM | 3 | 103 | 108 | -0.42 (-0.85, 0.00) | 0.05 | 58 |  |  |
|  | Hyperlipidemia | 1 | 38 | 35 | -0.74 (-1.21, -0.26) | 0.002 | - | 9.34 | 0.03 |
|  | Mean age |  |  |  |  |  |  |  |  |
|  | <45 years | 2 | 48 | 48 | 0.17 (-0.23, 0.57) | 0.41 | 0 |  |  |
|  | ≥45 years | 4 | 141 | 143 | -0.50 (-0.84, -0.16) | 0.004 | 50 | 6.30 | 0.01 |
|  | Mean BMI |  |  |  |  |  |  |  |  |
|  | <30 kg/m^2 | 3 | 113 | 115 | -0.46 (-0.90, -0.01) | 0.04 | 64 |  |  |
|  | ≥30 kg/m^2 | 3 | 76 | 76 | -0.12 (-0.70, 0.46) | 0.68 | 69 | 0.80 | 0.37 |
|  | ROB judgement |  |  |  |  |  |  |  |  |
|  | Low | 1 | 28 | 28 | -0.67 (-1.20, -0.13) | 0.02 | - |  |  |
|  | Unclear | 2 | 79 | 79 | -0.68 (-1.00, -0.35) | <0.0001 | 0 |  |  |
|  | High | 3 | 82 | 84 | 0.10 (-0.21, 0.40) | 0.53 | 0 | 13.48 | 0.001 |
|  | Jadad scale |  |  |  |  |  |  |  |  |

**Supplementary Table 9.** Cont.

| Effect size | Subgroups | Trial count | Intervention group size | Control group size | SMD (95% CI) | P for overall effect | I^2^ in subgroup (%) | Chi-square | P for subgroup differences |
| --- | --- | --- | --- | --- | --- | --- | --- | --- | --- |
| MDA* | Low | 2 | 61 | 63 | 0.13 (-0.22, 0.48) | 0.47 | 0 |  |  |
|  | High | 4 | 128 | 128 | -0.55 (-0.84, -0.25) | 0.0003 | 26 | 8.33 | 0.004 |
|  | Assignment |  |  |  |  |  |  |  |  |
|  | Crossover | 2 | 48 | 48 | 0.17 (-0.23, 0.57) | 0.41 | 0 |  |  |
|  | Parallel | 4 | 141 | 143 | -0.50 (-0.84, -0.16) | 0.004 | 50 | 6.30 | 0.01 |

*Effect measure was using standard mean difference. Abbreviation: MDA, malondialdehyde; MetS, metabolic syndrome; T2DM, type 2 diabetes; CI, confidential intervals; MD, mean difference; SMD, standard mean difference; ROB, risk of bias; RS, resistant starch.

**Supplementary Table 10.** Sensitivity Analysis Studies of Non-high Risk-of-bias Judgement

| Effect size | Trial count | Intervention group size | Control group size | MD/SMD (95% CI) | P for overall effect | I^2^ (%) |
| --- | --- | --- | --- | --- | --- | --- |
| Anthropometric parameters |  |  |  |  |  |  |
| BW (kg) | 7 | 249 | 249 | -1.44 (-3.66, 0.78) | 0.20 | 97 |
| BMI (kg/m^2^) | 6 | 211 | 210 | -0.62 (-1.28 0.04) | 0.06 | 95 |
| WC (cm) | 6 | 258 | 246 | -2.58 (-4.71, -0.45) | 0.02 | 52 |
| HC (cm) | 3 | 148 | 147 | -1.84 (-2.03, -1.64) | <0.00001 | 0 |
| Waist-to-hip ratio | 3 | 164 | 162 | -0.02 (-0.04, 0.00) | 0.05 | 39 |
| FM (kg) | 3 | 165 | 164 | -1.55 (-3.80, 0.71) | 0.18 | 97 |
| Body fat percentage (%) | 5 | 201 | 200 | -1.07 (-2.51, 0.36) | 0.14 | 93 |
| SBP (mmHg) | 5 | 196 | 196 | -1.93 (-4.60, 0.75) | 0.16 | 42 |
| DBP (mmHg) | 5 | 196 | 196 | -2.24 (-4.53, 0.05) | 0.06 | 58 |
| Glycemic profiles |  |  |  |  |  |  |
| FBG (mmol/L) | 12 | 314 | 322 | 0.01 (-0.14, 0.15) | 0.92 | 23 |
| FINS (μU/mL) | 9 | 295 | 298 | -2.67 (-3.83, -1.51) | <0.00001 | 86 |
| HbA1c (%) | 3 | 85 | 86 | -0.16 (-0.34, 0.03) | 0.11 | 26 |
| HOMA-IR | 7 | 254 | 255 | -0.66 (-1.04, -0.29) | 0.0005 | 71 |
| HOMA-β# | 3 | 49 | 49 | -1.08 (-22.76, 0.60) | 0.21 | 92 |
| Lipid profiles |  |  |  |  |  |  |
| TG (mmol/L) | 9 | 315 | 316 | -0.09 (-0.38, 0.20) | 0.53 | 88 |
| TC (mmol/L) | 9 | 315 | 316 | -0.21 (-0.35, -0.08) | 0.002 | 38 |
| HDL-C (mmol/L) | 9 | 315 | 316 | 0.05 (0.00, 0.09) | 0.03 | 52 |
| LDL-C (mmol/L) | 8 | 287 | 288 | -0.16 (-0.27, -0.05) | 0.005 | 29 |
| Inflammatory factors |  |  |  |  |  |  |
| hs-CRP^*^ | 3 | 85 | 90 | -0.23 (-0.52, 0.07) | 0.14 | 0 |
| TNF-α^*^ | 5 | 218 | 221 | -0.73 (-1.06, -0.40) | <0.0001 | 61 |
| IL-6 (pg/mL) | 3 | 164 | 166 | -0.19 (-0.47, 0.10) | 0.20 | 54 |
| Oxidative stress indicators |  |  |  |  |  |  |
| SOD^*^ | 5 | 107 | 107 | 0.33 (0.06, 0.60) | 0.02 | 0 |
| MDA^*^ | 3 | 107 | 107 | -0.67 (-0.95， -0.40) | <0.0001 | 0 |

^*^Effect measure was using standard mean difference. Abbreviation: BW, body weight; BMI, body mass index; WC, waist circumference; HC, hip circumference; WHR, waist-to-hip ratio; FM, fat mass; DBP, diastolic blood pressure; SBP, systolic blood pressure; FBG, fasting blood glucose; FINS, fasting insulin; HbA1c, glycated hemoglobin; HOMA-IR, homeostatic model assessment of insulin resistance; HOMA-β, homeostatic model assessment of beta-cell function; TG, triglycerides; TC, total cholesterol; HDL-C, high-density lipoprotein cholesterol; LDL-C, low-density lipoprotein cholesterol; hs-CRP, high-sensitivity C-reactive protein; TNF-α, tumor necrosis factor-alpha; IL-6, interleukin-6; MDA, malondialdehyde; SOD, superoxide dismutase.

**Supplementary Table 11.** Begg's and Egger's Test for Publication Bias

| Effect size | Begg's test | Egger's test |
| --- | --- | --- |
| Anthropometric parameters |  |  |
| BW | 0.251 | 0.363 |
| BMI | 0.251 | 0.111 |
| WC | 1.000 | 0.007 |
| HC | 0.308 | 0.133 |
| Waist-to-hip ratio | 0.734 | 0.894 |
| FM | 1.000 | 0.618 |
| Body fat percentage | 0.734 | 0.603 |
| SBP | 0.902 | 0.063 |
| DBP | 0.902 | 0.046 |
| Glucemic profiles |  |  |
| FBG | 0.488 | 0.033 |
| FINS | 0.304 | 0.329 |
| HbA1c | 0.027 | 0.076 |
| HOMA-IR | 0.592 | 0.407 |
| HOMA-β | 1.000 | 0.816 |
| Lipid profiles |  |  |
| TG | 0.304 | <0.001 |
| TC | 0.150 | 0.894 |
| HDL-C | 1.000 | 0.061 |
| LDL-C | 0.087 | 0.054 |
| Inflammatory factors |  |  |
| hs-CRP | 1.000 | 0.142 |
| TNF-α | 1.000 | 0.238 |
| IL-6 | 0.462 | 0.389 |
| Oxidative stress indicators |  |  |
| SOD | 0.221 | 0.069 |
| MDA | 0.707 | 0.375 |

The results showed P value of tests. Abbreviation: BW, body weight; BMI, body mass index; WC, waist circumference; HC, hip circumference; WHR, waist-to-hip ratio; FM, fat mass; DBP, diastolic blood pressure; SBP, systolic blood pressure; FBG, fasting blood glucose; FINS, fasting insulin; HbA1c, glycated hemoglobin; HOMA-IR, homeostatic model assessment of insulin resistance; HOMA-β, homeostatic model assessment of beta-cell function; TG, triglycerides; TC, total cholesterol; HDL-C, high-density lipoprotein cholesterol; LDL-C, low-density lipoprotein cholesterol; hs-CRP, high-sensitivity C-reactive protein; TNF-α, tumor necrosis factor-alpha; IL-6, interleukin-6; MDA, malondialdehyde; SOD, superoxide dismutase.
